# Highly stable and porous porphyrin-based zirconium and hafnium phosphonates – electron crystallography as an important tool for structure elucidation†
†Electronic supplementary information (ESI) available: Experimental procedures, analytical data including NMR spectroscopy, Pawley fits, TG, IR, microscopy and sorption measurements. CCDC 1831844. For ESI and crystallographic data in CIF or other electronic format see DOI: 10.1039/c8sc01533c


**DOI:** 10.1039/c8sc01533c

**Published:** 2018-05-28

**Authors:** Timo Rhauderwiek, Haishuang Zhao, Patrick Hirschle, Markus Döblinger, Bart Bueken, Helge Reinsch, Dirk De Vos, Stefan Wuttke, Ute Kolb, Norbert Stock

**Affiliations:** a Institut für Anorganische Chemie , Christian-Albrechts-Universität , Max-Eyth Straße 2 , D-24118 Kiel , Germany . Email: nstock@ac.uni-kiel.de; b Institute of Inorganic Chemistry and Analytical Chemistry , Johannes Gutenberg-University Mainz , Duesbergweg 10-14 , D-55128 Mainz , Germany . Email: kolb@uni-mainz.de; c Department of Chemistry and Center for NanoScience (CeNS) , University of Munich (LMU) , Butenandtstraße 5-13 , D-81377 Munich , Germany; d Centre for Surface Chemistry and Catalysis , KU Leuven , Celestijnenlaan 200f Box 2461 , B-3001 Leuven , Belgium

## Abstract

The Ni-metallated porphyrin-based tetraphosphonic acid (Ni-tetra(4-phosphonophenyl)porphyrin, Ni-H_8_TPPP) was used for the synthesis of highly porous metal phosphonates containing the tetravalent cations Zr^4+^ and Hf^4+^. The compounds were thoroughly characterized regarding their sorption properties towards N_2_ and H_2_O as well as thermal and chemical stability. During the synthesis optimization the reaction time could be substantially decreased under stirring from 24 to 3 h in glass vials. M-CAU-30, [M_2_(Ni-H_2_TPPP)(OH/F)_2_]·H_2_O (M = Zr, Hf) shows exceptionally high specific surface areas for metal phosphonates of *a*_BET_ = 1070 and 1030 m^2^ g^–1^ for Zr- and Hf-CAU-30, respectively, which are very close/correspond to the theoretical values of 1180 and 1030 m^2^ g^–1^. CAU-30 is always obtained as mixtures with one mol ZrO_2_/HfO_2_ per formula unit as proven by TEM, electron diffraction, TG and elemental analysis. Hence experimentally derived specific surface areas are 970 and 910 m^2^ g^–1^, respectively. M-CAU-30 is chemically stable in the pH range 0 to 12 in HCl/NaOH and thermally up to 420 °C in air as determined by variable-temperature powder X-ray diffraction (VT-PXRD). The crystal structure of M-CAU-30 was determined by combining electron diffraction tomography for structure solution and powder X-ray diffraction data for the structure refinement. The crystal structure consists of chains of corner sharing MO_6_ octahedra interconnected by the partly deprotonated linker molecules Ni-H_2_TPPP^6–^. Thus 1D channels with pore diameters of 1.3 × 2.0 nm are formed. The redox activity of Zr-CAU-30 was investigated by cyclic voltammetry resulting in a reversible redox process at a half-wave potential of *E*_1/2_ = –0.649 V.

## Abstract 2

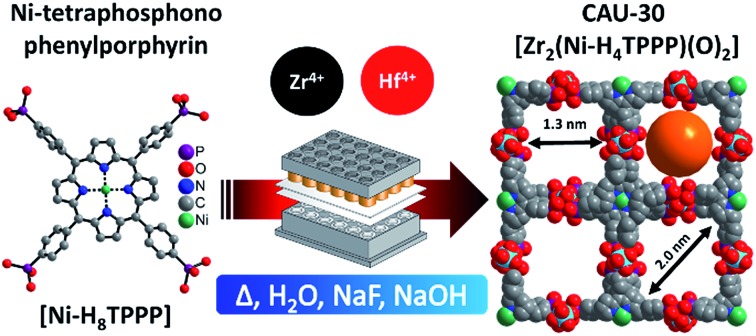
A highly porous and stable Zr-MOF containing a planar porphyrin-based tetraphosphonic acid was synthesized and characterized regarding its sorption properties and chemical stability.

## Introduction

Metal organic frameworks (MOFs) are an intensively investigated class of materials. Due to their modular structure they allow one to tune their pore size and pore surface properties, which in turn leads to a large range of chemical and physical properties.^1^–^4^ Although MOFs have been intensively investigated in applications such as gas storage and separation, drug delivery or catalysis^1^,^3^,^5^–^7^ their stability is often a limitation for most applications.^8^

The use of tri- and tetravalent cations often leads, especially for the metals Al, Cr and Zr to highly thermally and chemically stable MOFs.^9^–^13^ The investigation of these compounds is frequently hampered by the challenging structure determination since only microcrystalline products are obtained. Electron diffraction, *i.e.* automated diffraction tomography (ADT)^14^,^15^ and rotation electron diffraction (RED),^16^ has been established as a powerful tool to elucidate crystal structures of nano crystalline materials within the last decade.^17^–^21^ For beam sensitive materials like organic compounds and MOFs,^20^ data acquisition under cryogenic conditions at liquid nitrogen temperature, is important to reduce electron beam damage.

The combination of tetracarboxylic acids with Zr^4+^ and Hf^4+^ ions has been shown to yield highly interesting and functional compounds such as NU-1000,^22^ PCN-222 (MOF-545)^23^,^24^ or Zr-PPn.^25^ The latter two materials are porphyrin-based Zr-MOFs exhibiting carboxylate and phenolate groups respectively and they are active as catalysts in Fe^III^/Fe^II^ reduction^13^ in the Heck-reaction^26^ or CO_2_ reduction.^25^ The stability of those MOFs can be explained by the high oxophilicity of the tetravalent cations.^9^ According to the HSAB theory,^11^,^27^ hard acids like Zr^4+^ and hard bases like carboxylate or phosphonate groups result in stable Zr–O bonds.^27^ Comparing metal carboxylates to metal phosphonates, the latter are often more stable due to the higher charge and the increased number of donor atoms.^28^ This is in line with the larger number of coordination modes observed in metal phosphonates, which often leads to dense, layered structures.^29^,^30^

First studies in the field of Zr-phosphates were carried out by Clearfield *et al.* in 1964 by the preparation of Zr(HPO_4_)_2_·H_2_O^31^ and the structural characterisation in 1969.^32^ The work on porous Zr-phophonates began with the investigation of organically pillared Zr-phophonates in 1983, which are structurally related to Zr(HPO_4_)_2_·H_2_O. In these compounds the pores are statistically distributed, formed due to separation of the Zr–P–O layers by organic molecules.^33^

The total number of porous metal phosphonates is limited to only a few dozen structures^28^,^34^–^37^ and up to now only eight porous, crystalline Zr-phosphonate and no Hf-phosphonate MOFs have been reported (Table S1†): [Zr_2_H_4_(O_3_PCH_2_)_2_(N_2_C_4_H_8_)_3_],^38^ UPG-1,^39^ Zrbtbp,^40^ CALF-31,^41^ ZrH_4_L^42^ and most recently SZ-1 to 3.^43^ The specific surface areas of the known Zr-phosphonate MOFs range between 10 and 793 m^2^ g^–1^ (CALF-31).^41^

**Fig. 1:**
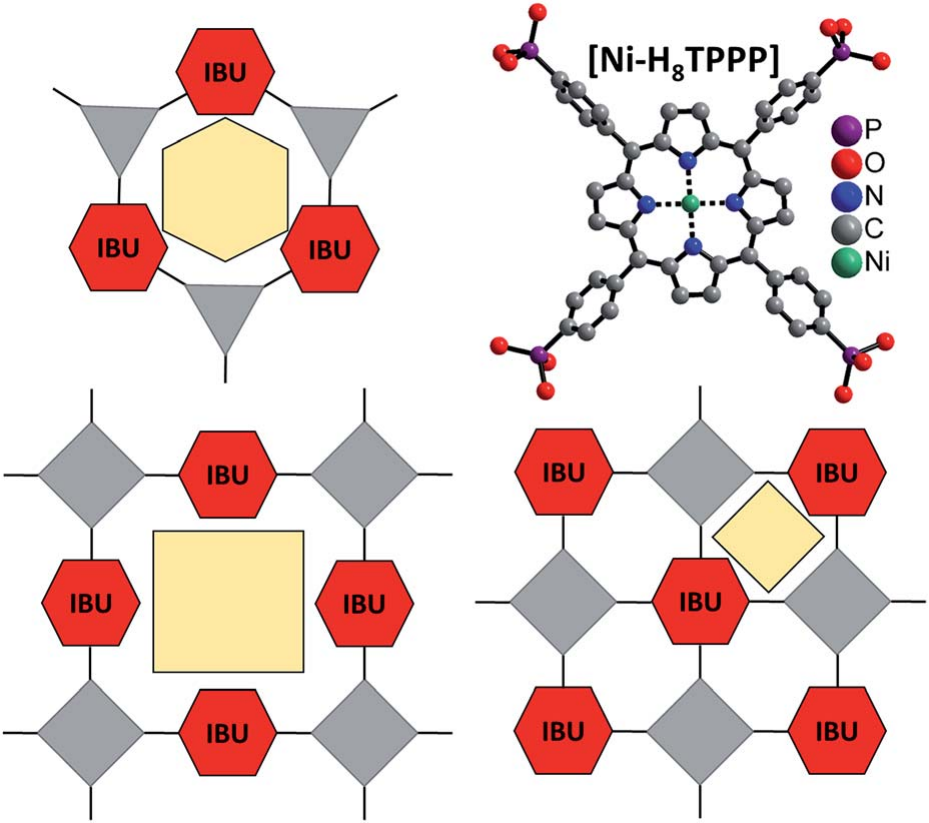
Concept for the design of porous Zr and Hf phosphonates with 1D IBUs (red hexagons) using rigid and planar tetratopic phosphonic acid as linker molecule (top, right). Top, left: scheme of known porous Zr triphosphonates with honeycomb structure and a 1D IBU (red hexagons).^39^,^40^ Bottom: possible connection of the 1D IBUs by planar tetraphosphonate ions which should lead to the formation of porous metal phosphonates. Grey triangles and squares represent tri- and tetraphosphonic acids and yellow polygons the schematic pore space.

In contrast to numerous porphyrin-based MOFs bearing carboxylate groups^44^,^45^ to date only one such MOF (CAU-29) containing phosphonate groups has been reported.^46^ After the first report of tetra(4-phosphonoethylphenyl)porphyrin, H_10_TPPP in 1995 (ref. 49) (Fig. 1) molecular structures such as the ester^47^,^48^ and different metalated compounds with di- and trivalent cations (Mn^3+^,^49^,^50^ Ni^2+^,^46^ Zn^2+^)^48^,^51^ have been reported. These molecules have been characterized in literature regarding their electrochemical^47^,^48^,^52^ and photochemical^48^,^52^,^53^ properties as well as in catalytic^49^,^50^ and self-assembling^54^,^55^ or -aggregation^56^,^57^ processes.

Comparison of porous Zr-phosphonates reveals preferred formation of linear inorganic building units (IBU) consisting of ZrO_6_ octahedra.^38^–^40^ In combination with rigid tritopic phosphonic acids, which turned out to suppress the layer motive, “honeycomb” like pores were mostly observed in porous Zr-phosphonates (Fig. 1).^39^,^40^ Applying this information to rigid tetratopic phosphonic acids two types of structures exhibiting pores of different size can be envisioned (Fig. 1). Therefore the combination of tetravalent cations with geometrically demanding phosphonic acids could lead to highly porous and exceptionally stable MOFs.^43^

Here we present our results which are based on the previously mentioned considerations. We have carried out a systematic investigation on Zr- and Hf-phosphonates using the square planar tetraphosphonic linker Ni-H_8_TPPP in hydrothermal reactions.

## Results and discussion

### Synthesis

The systematic study of the system Ni-H_8_TPPP/M^4+^/NaF/NaOH (M = Zr^4+^, Hf^4+^) using high-throughput (HT) methods resulted in highly porous and stable metal phosphonates (Fig. S5a†). The product formation depends mainly on the presence of F^–^ ions and the pH value of the reaction mixture (pH_start_ = 7). In case of Zr- and Hf-CAU-30 a molar linker : metal : NaF : NaOH ratio of 1 : 2 : 60 : 8 resulted in highly crystalline products (1 equivalent corresponds to 5.3 × 10^–3^ mmol). The syntheses can be scaled up by at least to a factor of 12. The initially used reaction time and temperature can be reduced from 24 to 3 h and 180 °C to 160 °C, respectively, when a glass reactor is employed and the reaction mixture is stirred (Fig. S5b†). Although F^–^ ions are present in the reaction mixture no reaction with the glass reactor was observed.

### Crystal structure

**Fig. 2:**
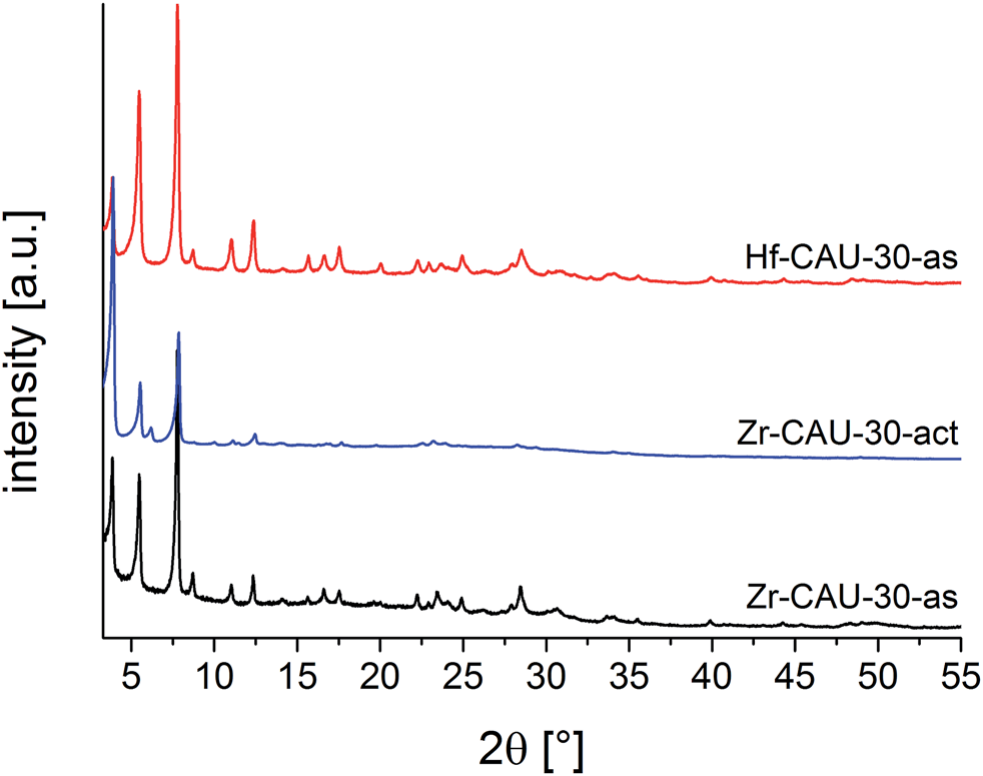
PXRD patterns of Zr-CAU-30-as, Zr-CAU-30-act and Hf-CAU-30-as.

Zr- and Hf-CAU-30 were obtained as microcrystalline powders. Therefore the structure of Zr-CAU-30 was solved employing automated electron diffraction tomography (ADT), while the structure was refined from the PXRD data of an activated (-act) sample, *i.e.* Zr-CAU-30-act (Fig. S7b,† Table 1). The PXRD patterns of the as-synthesized (-as) form Zr- and Hf-CAU-30-as are compared with the one of Zr-CAU-30-act in Fig. 2. Activation was carried out at 250 °C under reduced pressure of 10^–2^ kPa in a 0.5 mm glass capillary. The differences in the relative intensities of Zr-CAU-30-as and -act are due to the loss of H_2_O molecules from the pores.

**Table 1:** Results of the Rietveld refinement of Zr-CAU-30-act (activated at 250 °C for 2 h under reduced pressure of 10^–2^ kPa) and the Pawley refinements of M-CAU-30-as (M = Zr, Hf)

	Zr-CAU-30-act	Zr-CAU-30-as	Hf-CAU-30-as
Method	Rietveld	Pawley	Pawley
Crystal system	Tetragonal	Tetragonal	Tetragonal
*a* [Å]	44.778(6)	45.121(5)	45.040(5)
*b* [Å]	44.778(6)	45.121(5)	45.040(5)
*c* [Å]	7.658(4)	8.090(2)	8.049(3)
*α* [°]	90	90	90
*β* [°]	90	90	90
*γ* [°]	90	90	90
*V* [Å^3^]	15 354(8)	16 470(5)	16 329(8)
Space group	*I*4_1_*cd*	*I*4_1_/*acd*	*I*4_1_/*acd*
GoF	2.0	0.72	0.94
*R*_wp_, *R*_bragg_ [%]	3.8, 0.5	3.3	3.8

The tetragonal cell indexed from the PXRD pattern of Zr-CAU-30-act is in line with the unit cell parameters of the as-synthesized form obtained from reconstruction of 3D electron diffraction data (Table 1). However, the extinction conditions indicate a lower symmetry for the activated compound. The amount of water in the pores has a strong influence on the relative intensities hence the activated sample was chosen for the structure refinement in *I*4_1_*cd* symmetry (Fig. S7b†). To prove the reversibility of the transformation from the activated (*I*4_1_*cd*) into the as-synthesized (*I*4_1_/*acd*) form, PXRD patterns of the activated form in an open capillary were recorded every five minutes over five days (Fig. S7d†). The changes in relative intensities upon hydration is best detected monitoring the 130 reflection. While in the as-synthesized compound only reflections *hk*0 with *h* = 2*n* are observed indicating the presence of an *a*-glide plane, additional reflection such as the 130 reflections are clearly seen in the PXRD pattern of the activated sample (Fig. S7d†).

### Structure solution by electron diffraction tomography

Needle-like crystals with different sizes (Fig. S6a†) could be observed for Zr-CAU-30-as. The diameters of the nanorods range from a few tens up to about 500 nm, and are therefore suitable for TEM experiments. The crystals are stable under cooling conditions using low beam illumination. Nano electron diffraction experiments show that the nanorods are highly crystalline.

Aggregates of spherical nanoparticles smaller than 5 nm with partial crystallinity were also observed by TEM as a minor secondary phase (Tables S5 and S6†). Crystals with a diameter of some hundred nanometres were selected for TEM experiments.

Cell parameters extracted from three-dimensional electron diffraction data describe a unit cell with *a* = 46.09 Å, *b* = 45.74 Å, *c* = 7.81 Å (Fig. S6b†), which are in line with the results obtained from PXRD (Fig. S7a† and Table 1). Systematic extinctions conditions with *h* + *k* + *l* = 2*n* for the *hkl* reflections indicated an *I*-centred Bravais lattice. The reflection conditions can be observed from 2D-cuts from the reconstructed lattice (Fig. S6c†) as following *h* = 2*n* and *k* = 2*n* for *hk*0; *k* = 2*n* and *l* = 2*n* for 0*kl*; 2*h* + *l* = 4*n* for *hkl* delivering the extinction rule *Iacd* associated with the tetragonal space group *I*4_1_/*acd* (no. 142). An ADT dataset collected in combination with electron beam precession from a single nanocrystal with a tilt range from –65 to +58 (Table 2) was used for crystal structure solution employing direct methods.

**Table 2:** Experimental parameters of electron diffraction dataset for structure solution of Zr-CAU-30-as in space group *I*4_1_/*acd*

Tilt range (°)	–65/+58
No. of total reflections	18 149
No. of independent reflections	2021
Reflection coverage (%)	99
Resolution (Å)	1.0
*R*_int_	0.344
Overall *U* (Å^2^)	0.023
Residual *R* (SIR2014)	0.173

The *ab initio* structure solution converged with a final residual *R* value of 0.173. The structure solution delivered a well-resolved Fourier potential map (Fig. S6d†), accompanying some extra potentials, which probably correspond to residual water molecules in the pores. The two strongest maxima (2.24 and 1.63 e^–^ Å^–3^) correspond to the two metal atoms Zr and Ni, respectively. The next maximum with 1.24 e^–^ Å^–3^ is consistent with the position of the P atom. The following peaks for O, N and C atoms were detected with a scattering potential range of 0.98 down to 0.49 e^–^ Å^–3^. One missing O atom located at P could only be detected from the difference Fourier map.

Structure refinement was accomplished from PXRD data of the activated compound (Zr-CAU-30-act).‡
‡Crystal data: Zr_2_O_21.1_P_4_C_44_N_4_Ni_1_, *M* = 1285.5 g mol^–1^, tetragonal, *a* = *b* = 44.7776(58), *b* = 15, *c* = 7.6581(39) Å and *α* = *β* = *γ* = 90°, *U* = 15 354(8) Å^3^, *T* = 298 K, space group *I*4_1_*cd* (no. 110), *Z* = 8. The final *R*_wp_ was 3.8% and the g of 2.0%. Starting with the structure model of the framework as determined from electron diffraction data, we set up a model by first converting the space group symmetry from *I*4_1_/*acd* to *I*4_1_*cd* using Powdercell^58^ and subsequent optimisation of the structure using the universal force field as implemented in Materials Studio.^59^ Due to the low number of reflections in combination with the very large unit cell only a partial Rietveld refinement could be carried out. Hence, for example the position of the porphyrin moiety was fixed and the phenylphosphonate fragments were treated as rigid bodies. The Zr–O bond lengths were restrained to literature values. More details are given in the Experimental section.

### Structure description

**Fig. 3:**
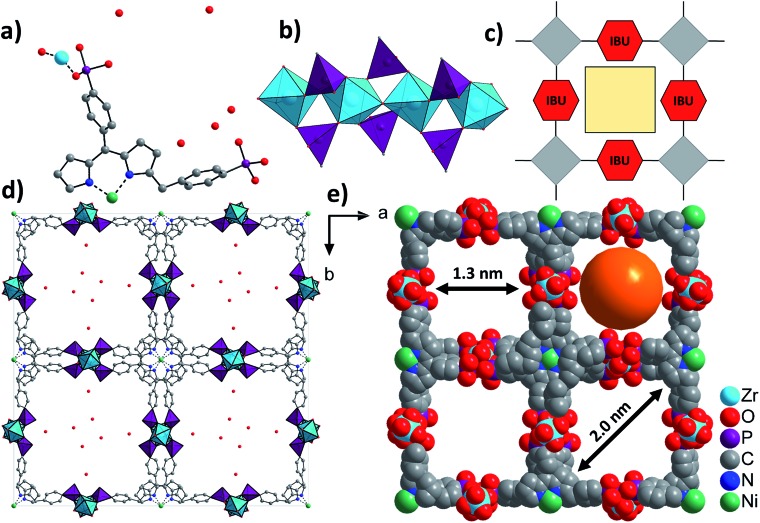
Structural representation of Zr-CAU-30, (a) the asymmetric unit, (b) the IBU consisting of chains of trans corner sharing ZrO_6_/ZrO_4_F_2_ octahedra further connected by PO_3_C tetrahedra to form 1D chains, view along [100], (c) schematic representation of a porous structure composed of square planar tetratopic linker and a 1D IBU according to Fig. 1, (d) the network structure of Zr-CAU-30 along [001] with H_2_O molecules in the pores and (e) space filling view along [001] and pore diameters of 1.3 and 2 nm, respectively, measured by taking the van der Waals radius of the atoms lining the pores into account (water molecules are omitted for clarity).

The asymmetric unit of Zr-CAU-30, [Zr_2_(Ni-H_2_TPPP)(OH/F)_2_]·23H_2_O, is shown in Fig. 3a and consists of one Zr^4+^ and one (OH^–^/F^–^) ion as well as a linker located on an centre of inversion. In addition water guest molecules are shown. The inorganic building unit (IBU) of Zr-CAU-30 consists of chains of trans corner sharing ZrO_6_/ZrO_4_F_2_ octahedra (Fig. 3b) where bridging is accomplished through μ-OH^–^/F^–^ ions. Each chain is interconnected to six other chains by the Ni-H_4_TPPP^4–^ linker ions and a 3D framework with 1D channels exhibiting pore diameters of 1.3 along the edge and 2.0 nm along the diagonal of the pore (Fig. 3d) are formed (Connolly surface Fig. S6e†). Furthermore each linker molecule is coordinated to eight Zr^4+^ ions (Fig. S6f†).

One of the two symmetry independent phosphonate groups is probably protonated and based on the donor–acceptor distance of 2.6(2) Å to a guest molecule in the pores a hydrogen bond can be anticipated. The presence of P-OH and μ-OH groups was further proven by IR spectroscopy. The coordination mode of the phosphonate and the hydrogenphosphonate group is each [2,110] employing the Harris notation.^29^ The Harris notation has the format [*A*,*XYZ*], the value *A* being the number of metal ions coordinated by the hydrogenphosphonate group and *X*, *Y*, *Z* the number of bonds each oxygen atom shares with a metal ion.^29^

The use of F^–^ ions in the synthesis of crystalline Zr-phosphonates has been previously shown to be highly beneficial.^39^,^41^ However the quantitative determination is often challenging. In this work, the fluoride content was determined by means of a fluoride-sensitive electrode. Before the analysis, the samples were digested by fusion with Na_2_CO_3_/K_2_CO_3_: [Zr_2_(H_2_TPPP)F_2_]·23H_2_O·ZrO_2_ calc.: 2.2%, found: 3.5% and [Hf_2_(H_2_TPPP)F_2_]·30H_2_O·HfO_2_ calc.: 1.8%, found: 3.9%. Considering a large standard deviation due to the low content of F^–^ in CAU-30 the values are in a reasonable range.

**Fig. 4:**
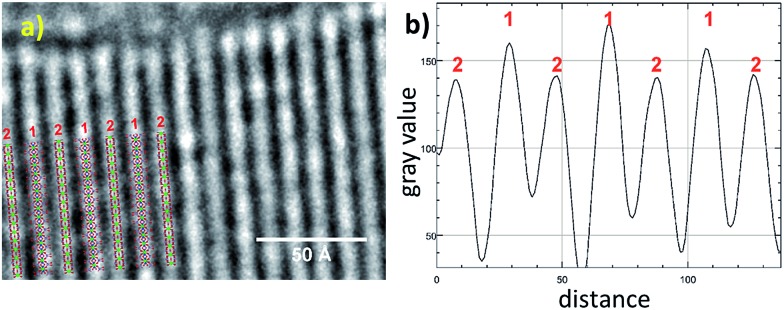
(a) High resolution TEM: reconstructed phase image of Zr-CAU-30-as recorded along [010] zone axis (red); (b) intensity profiles of the electron density 1 and 2 in (a).

To further confirm the crystal structure additional high-resolution transmission electron microscopy (HRTEM) measurements were carried out to visualize structural details of Zr-CAU-30-as. Fig. 4 shows a reconstructed phase image based on the analysis of a focal series of 20 images, the sections of the crystal structure with Zr, Ni, P and O atoms, and the intensity profile. A direct comparison with the crystal structure of Zr-CAU-30 viewed along [010] reveals already a high resemblance. The crystal structure viewed along the crystallographic *b* axis shows two columns, one containing Zr and Ni clusters (marked as 1) and another only with Zr clusters (marked as 2). The enhanced density of column 1 is evident in the extracted intensity profile.

### Particle morphology and composition by SEM and TEM

**Fig. 5:**
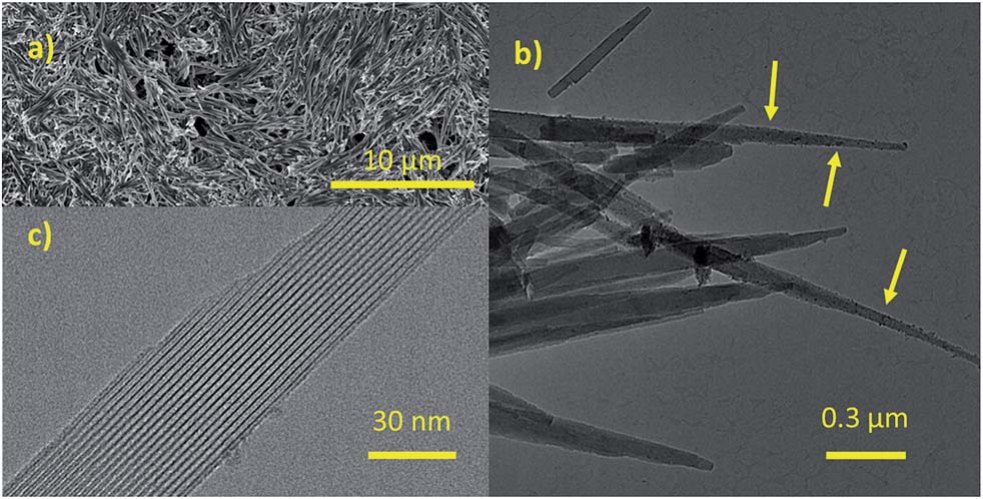
Results of the SEM and TEM measurements performed on Zr-CAU-30. (a) SEM image of Zr-CAU-30, (b) TEM image of needles of Zr-CAU-30 with small spherical particles of ZrO_2_ on the surface of the needles (marked with arrows), (c) a detailed TEM image of one needle.

To determine the morphology of the Zr-CAU-30 crystals, SEM measurements were performed. The results are depicted in micrograph (a) in Fig. 5. The MOF crystallizes in form of needles that are up to 6 μm long and 200 nm broad. In subsequent TEM measurements shown in the micrographs (b) and (c) the crystallinity of the sample was confirmed: the needles in micrograph (c) depict a visible periodicity perpendicular to the growth direction of the needles. The Fourier-transform of a diffraction pattern was used to determine the lattice parameters of the respective crystals (Fig. S6g†). The resulting spots in the reciprocal space correspond to lattice distances of 2.35 nm, 1.18 nm and 0.77 nm respectively and are well in agreement with the first (020 at *d* = 2.24 nm), fourth (040 at *d* = 1.12 nm) and eleventh (0.75 nm, 060) reflection of the PXRD pattern. As seen in (b), there is a secondary phase present in the sample in form of very small spherical particles (3–5 nm) on the surface of the needles. Further characterization revealed that this phase consist of monoclinic ZrO_2_ (details are shown in the ESI Section 6†). The SEM, TEM and EDX results for M-CAU-30 (M = Zr, Hf) are accordingly given in the ESI Section 6.†

### Sorption properties

**Fig. 6:**
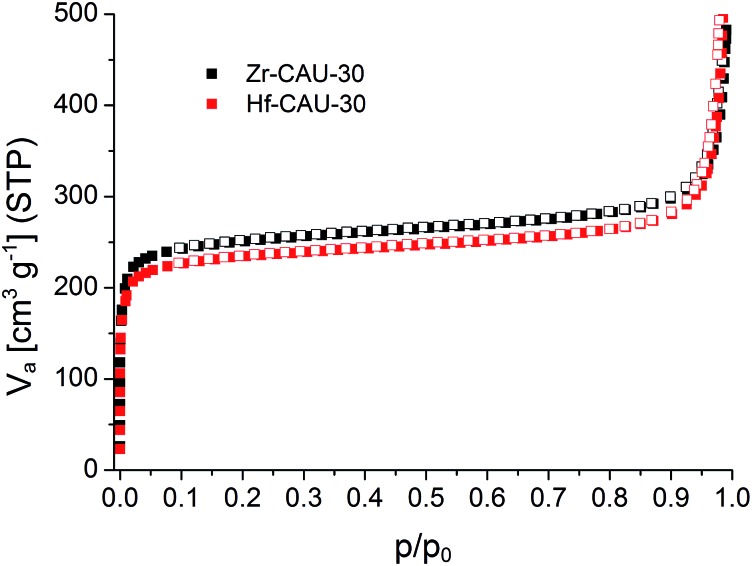
N_2_-sorption isotherms of M-CAU-30 (M = Zr (black), Hf (red)) measured at 77 K.

Sorption experiments were carried out for both title compounds. Prior to the measurements all samples were activated at 170 °C under vacuum (10^–2^ kPa) for 16 h. Subsequently sorption experiments using N_2_ at 77 K (Fig. 6) and H_2_O at 298 K (Fig. S8†) as adsorptives were performed. The specific surface areas were determined using the BET method and applying the method of Rouquerol.^60^,^61^ Thus, the BET equation has been fitted between *p*/*p*_0_ = 5.223 × 10^–6^ – 0.103 and 6.740 × 10^–5^ – 0.078 for Zr- and Hf-CAU-30, respectively. Micropore volumes *V*_m_ were determined by using the amount of adsorbed N_2_ at the relative pressure *p*/*p*_0_ = 0.5 (Table 3). Both compounds are stable towards activation and sorption experiments as proven by PXRD measurements (Fig. S9†). The N_2_ measurements led to a Type-1 isotherm as expected for microporous materials.^61^ Experimental specific surface areas of *a*_BET_ = 970 (Zr) and 910 (Hf) m^2^ g^–1^ were obtained (Table 3). The H_2_O sorption measurements at 298 K (Fig. S8†) show uptakes of 250 (Zr) and 340 (Hf) mg g^–1^.

**Table 3:** Results of the N_2_ sorption experiments at 77 K of M-CAU-30 (M = Zr, Hf). The micropore volume was calculated at *p*/*p*_0_ = 0.5. The theoretical specific surface areas were calculated using the program Materials Studio V5 and were each calculated for the pure framework and the framework with one mol MO_2_ per formula unit (framework + MO_2_)

	Zr-CAU-30 framework + ZrO_2_	Zr-CAU-30 pure framework	Hf-CAU-30 framework + HfO_2_	Hf-CAU-30 pure framework
N_2_ (*a*_BET_), [m^2^ g^–1^]	970	1070	910	1030
*a*_BET_ (theory)	980	1180	890	1030
N_2_ (*V*_m_), [cm^3^ g^–1^]	0.41		0.38	
H_2_O (upt.), [mg g^–1^]	250		340	

The theoretical specific surface area of Zr-CAU-30 was calculated using the program Materials Studio V5.^59^ Employing a probe molecule of 1.8 Å radius the maximum specific surface area was calculated to be 1180 m^2^ g^–1^. The difference of *ca.* 200 m^2^ g^–1^ to the measured specific surface area is reasonable due to the presence of crystalline ZrO_2_ species (ESI, Section 6†) on the particles of Zr-CAU-30. Taking the impurity of one ZrO_2_ per formula unit, as determined from the TG and elemental analysis, into account the specific surface area of the single phase CAU-30 amounts to 1070 and 1030 m^2^ g^–1^.

### Thermal and chemical stability

To investigate the thermal stability thermogravimetric (TG) measurements of all samples and variable temperature (VT) PXRD studies were carried out.

**Fig. 7:**
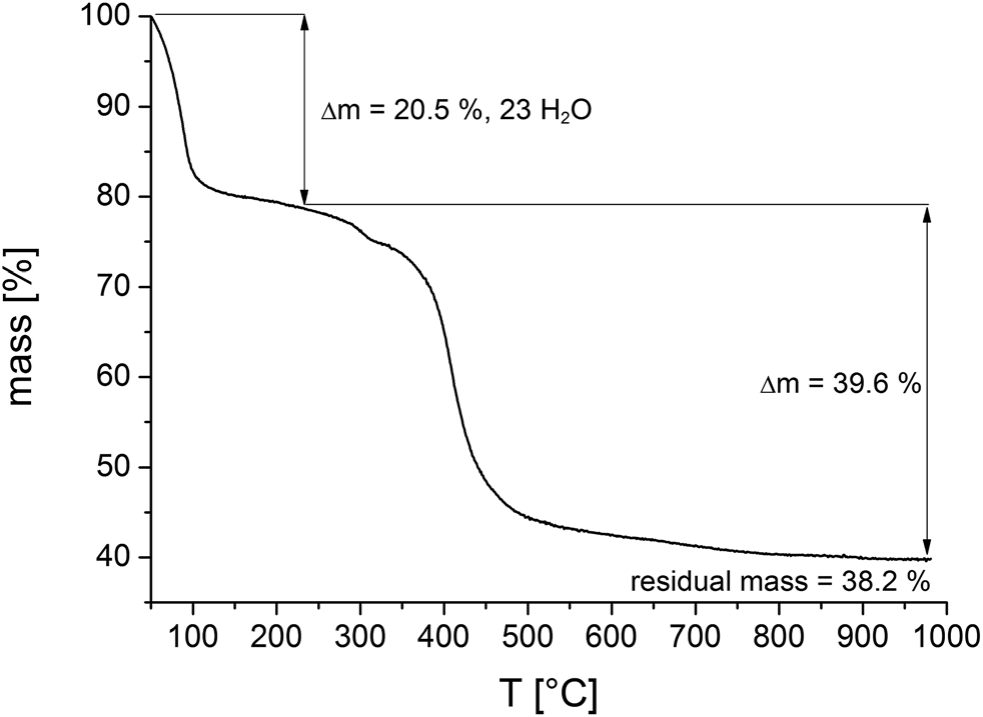
Thermogravimetric curve of a sample of Zr-CAU-30 (Table 3). Comparison of calculated and theoretical weight losses in combination with the results of the SEM and TEM measurements allow to quantify the amount of ZrO_2_ in the reaction product as one ZrO_2_ per formula unit.

The results of the TG measurements (Fig. 7 and S10, Table S3†) show two characteristic steps of weight loss, respectively. The loss up to 120 °C is assigned to the evaporation of physisorbed water molecules. Above 400 °C the decomposition of the compounds take place and different M^IV^Ni^II^-phosphates (M = Zr, Hf) are formed as the final product, which could partially be identified by PXRD measurements (Fig. S11†). In general, the results of the TG analysis fit well with the ones of the elemental analyses. Slight differences in the amounts of occluded solvent molecules are due to the storage of the samples prior to the measurements. The comparison of the calculated and the measured residual mass of the title compounds confirm the presence of inorganic impurities as identified by TEM and EDX measurements to be the monoclinic phases of ZrO_2_ and HfO_2_, respectively (Tables S5 and S6†). The sample contains one mol ZrO_2_ per formula unit of CAU-30.

**Fig. 8:**
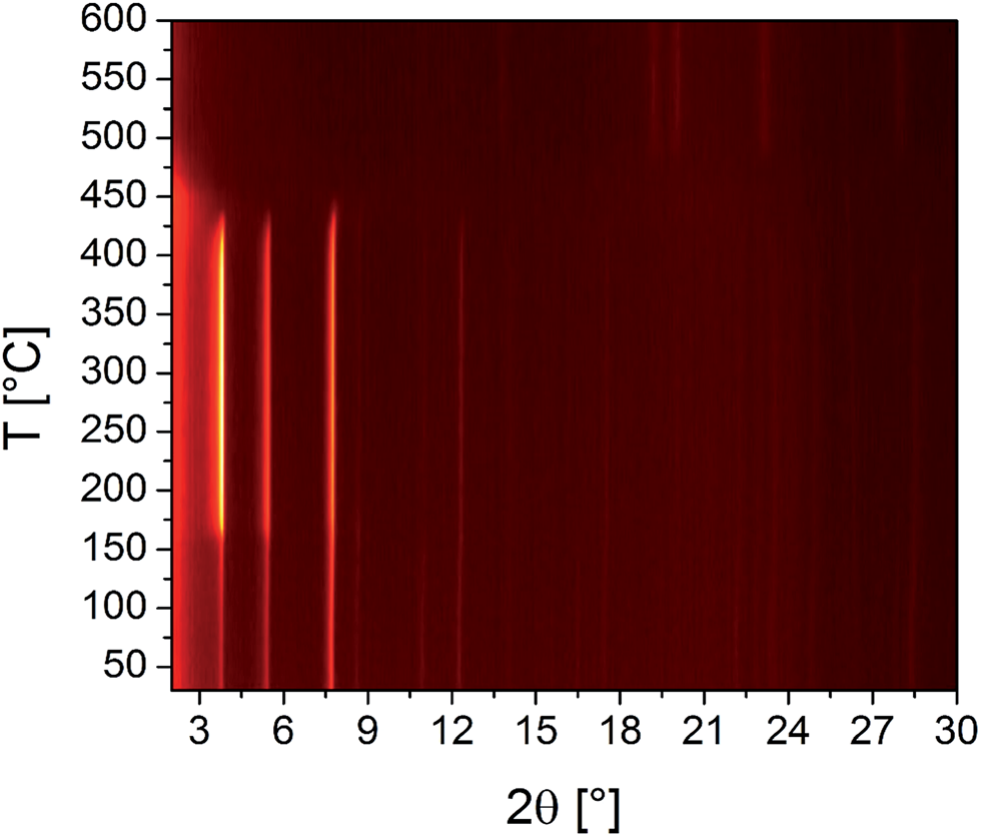
Results of the VT-PXRD study of Zr-CAU-30 (Cu-Kα_1_ radiation) measured in an open quartz capillary (0.5 mm).

The results of the VT-PXRD studies of Zr-CAU-30 (Fig. 8) and Hf-CAU-30 (Fig. S12†) fit well to the results of the TG measurement. Apart from the decomposition of the framework at 400 °C, no phase transformations are observed. Up to 150 °C, the relative reflection intensities change strongly which is in line with the loss of physisorbed water molecules and the activated form, *i.e.* Zr-CAU-30-act is formed. Identical results were observed for Hf-CAU-30 (Fig. S12†). The small differences compared to the TG measurements are due to the confinement of the sample in a capillary during the VT-PXRD measurement.

**Fig. 9:**
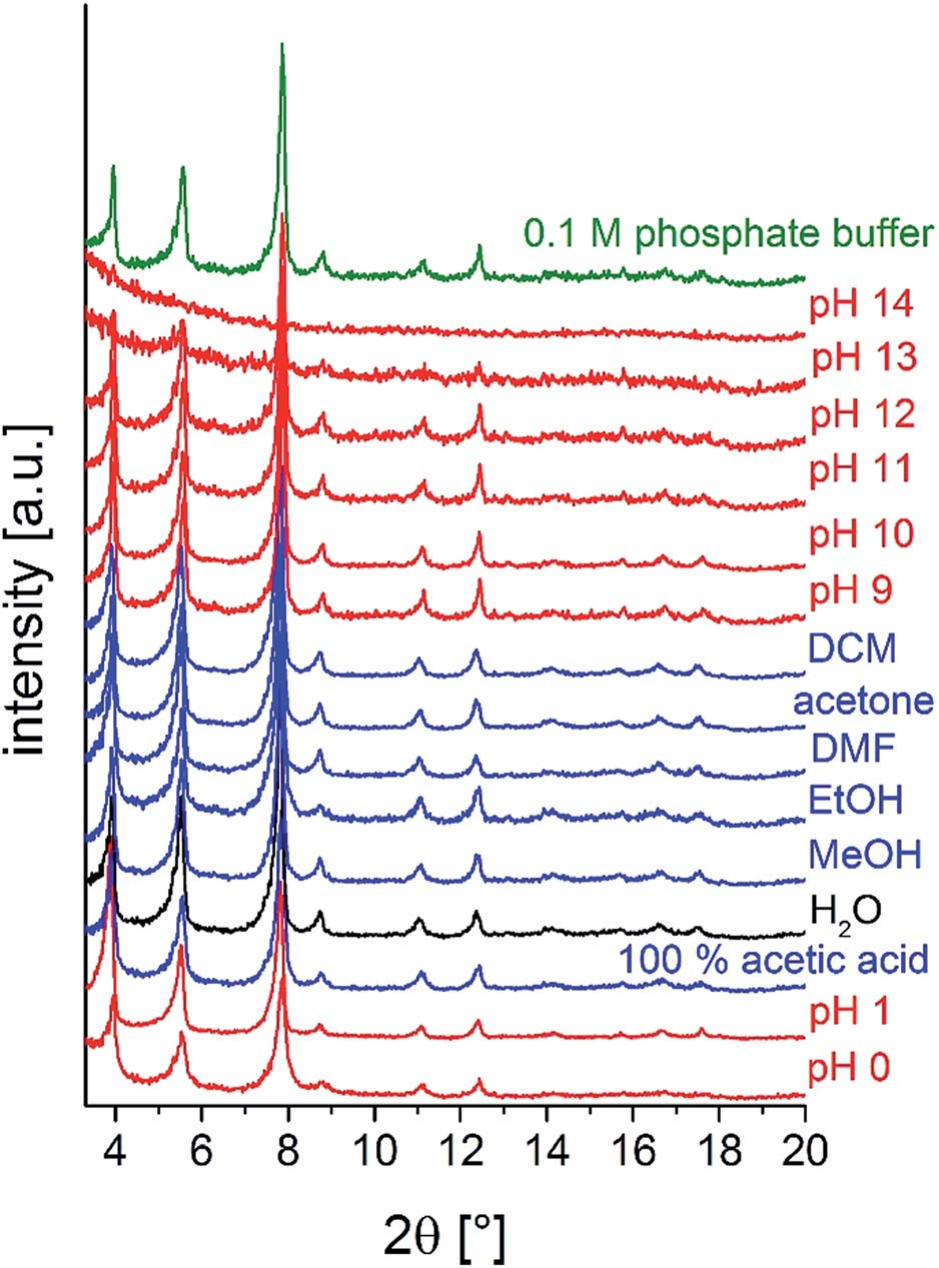
Chemical stability of Zr-CAU-30 in different solvents (24 h, stirring at room temperature).

To investigate the chemical stability, M-CAU-30 (M = Zr, Hf) was exposed to different solvents (HCl (pH 0–7), NaOH (pH 7–14), 100% acetic acid, H_2_O, MeOH, EtOH, acetone, DMF, dichloromethane and 0.1 M phosphate buffer) at RT for 24 h under stirring. After the treatment the samples were isolated by filtration and measured by PXRD. The results of the chemical stability tests (Fig. 9 and S13†) reveal that M-CAU-30 is stable in all tested organic solvents as well as in a pH range between 0 and 12 (aqueous HCl/NaOH solutions). Remarkably, M-CAU-30 is also stable in 0.1 M phosphate buffer (pH 7).

### Spectroscopy

IR spectra of the title compounds and the Ni-H_8_TPPP linker are shown in Fig. S14a.† The assignment of the bands are given in Table S4† in detail. The characteristic bands for M-CAU-30 (M = Zr, Hf) are identical and compared with the ones of the free linker Ni-H_8_TPPP.

In the following discussion the position of the bands of the free linker Ni-H_8_TPPP are given in brackets, behind the ones of the MOF material. Vibrations of the porphyrin moiety like the *ν*(C=C), *δ*(C=C, C=N), *ν*(C=N), *γ*(C–H) and *δ*(C–H, N–H) are observed at 1560, 1390, 1350, 800 and 737 cm^–1^, respectively.^62^ Characteristic bands of the hydrogenphosphonate (phosphonic acid) group are for example the *ν*(P–O(OH)) and *ν*(P–C) vibrations at 1634 (1606) and 1501 (1480) cm^–1^,^63^ but the band at 1634 cm^–1^ is mainly due to the presence of water molecules (*δ*_s_ vibration) in the non-activated sample. The *ν*(P=O) and *ν*(P–O) bands are observed at 1234 (1223), 1141 (1136) and 966 (921) cm^–1^. Furthermore *γ*(P–C) and *δ*(P(OR)_3_) deformation vibrations are observed at 717 (704) and 588 (567) cm^–1^.^10^,^11^,^64^

**Fig. 10:**
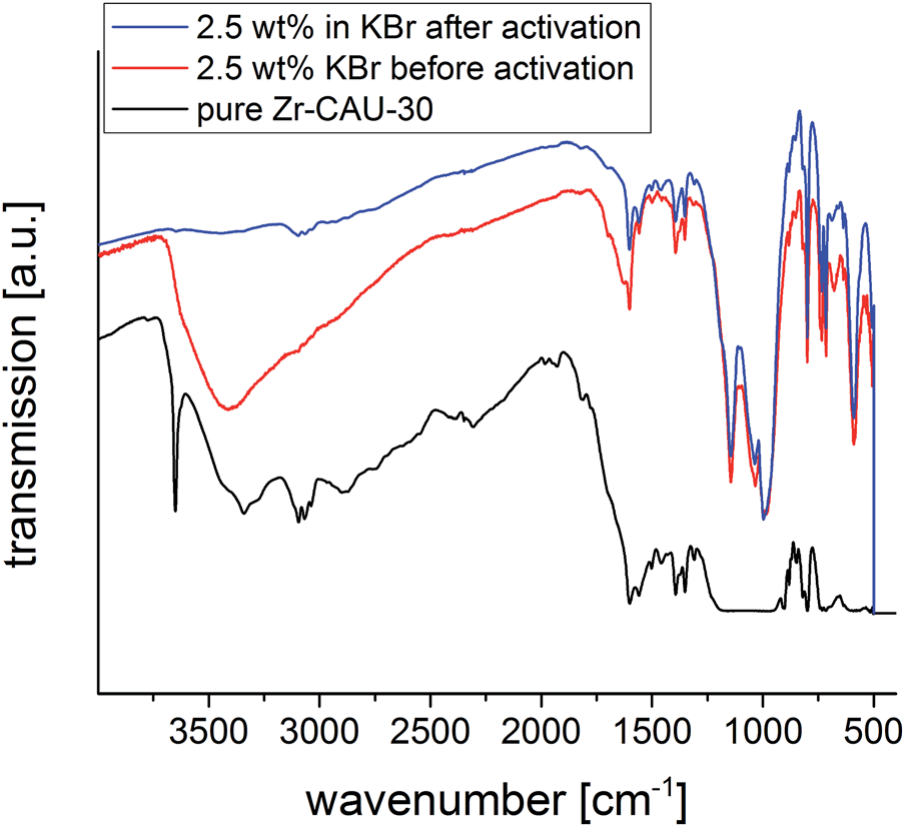
IR spectra of Zr-CAU-30 with 2.5 wt% KBr before (red) and after (blue) activation at 200 °C as well as the pure sample (black).

More insight into the proton topology of Zr-CAU-30 was gathered from FTIR spectra recorded after an *in situ* thermal evacuation (200 °C) of the material (Fig. 10). Two samples were investigated, the pure Zr-CAU-30 material, and the same diluted as 2.5 wt% in dry KBr. The spectrum of the latter corresponds well with that of the non-evacuated sample, featuring multiple prominent bands corresponding to *ν*(P–O) (1143 cm^–1^; 997 cm^–1^), *γ*(P–C) (715 cm^–1^) and *δ*(P(OR)_3_) (590 cm^–1^). Other bands attributable to vibrational modes in the linker are found at 734 cm^–1^, 799 cm^–1^, 1350 cm^–1^, 1390 cm^–1^ and 1560 cm^–1^. The broad band centered at 3400 cm^–1^ in the non-evacuated sample, attributable to water and hydrogen-bonded μ-OH-groups, fully disappears upon activation. In its place, a small OH-band at approximately 3640 cm^–1^ is observed (Fig. 10, blue), which correspond to the μ-OH groups bridging the metal ions. In the spectrum of the pure Zr-CAU-30 sample (Fig. 10, black), this band is clearly pronounced, and in fact three distinct vibrations, a prominent one at 3650 cm^–1^, are observed. Additionally, a broad band in the 2240–2440 cm^–1^ region can be observed. The presence of both vibrational modes is indicative of P-OH groups in the material. Nevertheless, it cannot be excluded that the much weaker *ν*(O–H) bands originate from μ-OH groups connecting the ZrO_6_-octahedra.

**Fig. 11:**
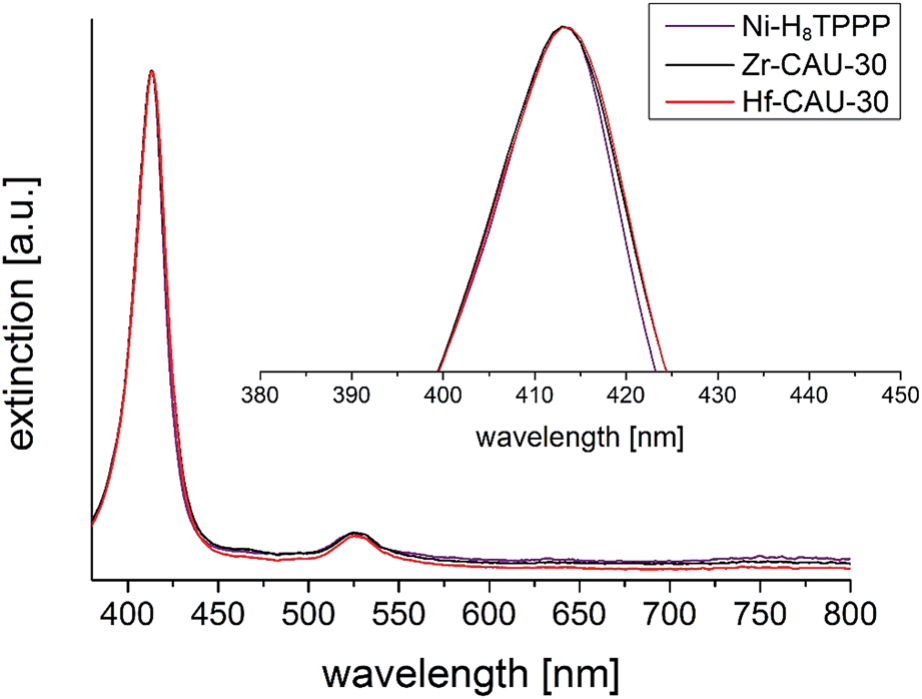
UV/vis spectra of M-CAU-30 (M = Zr, Hf) compared with the UV/vis spectrum of the free linker Ni-H_8_TPPP. The absorption maximum (Soret-band) of the Ni-H_8_TPPP linker was in all cases found at 413 nm, furthermore the Q1-band was found at 526 nm.

UV/vis spectroscopy was employed to prove the metalation of the porphyrin moiety after the hydrothermal reaction. The pure linker Ni-H_8_TPPP as well as the two MOFs exhibited nearly identical positions for the Soret- and Q1-band at 413 and 526 nm, respectively (Fig. 11). Thus the high stability of the metalated porphyrin linker under hydrothermal conditions at 180 °C for 48 h is confirmed.

### Redox activity

**Fig. 12:**
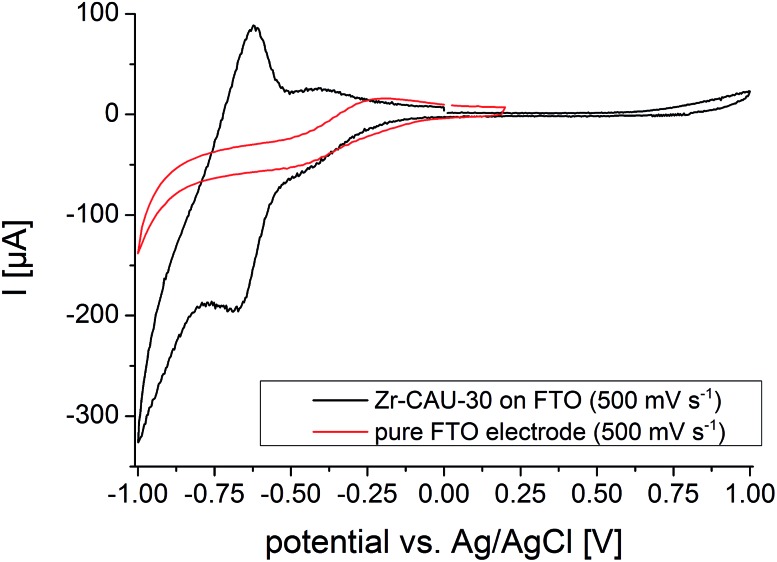
CV curves of Zr-CAU-30 on FTO (black) and the pure FTO electrode (red) between –1 and 1 V and a scan rat of 500 mV s^–1^.

Cyclic voltammograms (CVs) of Zr-CAU-30 were measured on a fluorine doped tin oxide (FTO) electrode in 0.1 M phosphate buffer (pH 7) with a Ag/AgCl type reference electrode and Pt as counter electrode. The complete CV between –1 and 1 V of Zr-CAU-30 (on FTO) in comparison with the pure FTO electrode is shown in Fig. 12. In this CV a reversible redox process at a half-wave potential of *E*_1/2_ = –0.649 V is observed which demonstrates the redox activity of Zr-CAU-30 under these conditions. In comparison to literature values of Ni-metalated^65^,^66^ and phosphonate groups bearing porphyrin derivatives^48^,^53^ this redox process at –0.649 V can likely be assigned to the reversible reduction of the porphyrin moiety. Furthermore, the cyclic stability was demonstrated by measuring 50 cycles (scan rate = 250 mV s^–1^); the first and the 50^th^ CV is shown in Fig. S22.† Applying different scan rates between 50 and 1000 mV s^–1^ reveals the expected behaviour of increasing currents with the scan rate (Fig. S23†).

## Conclusions

In summary, we have demonstrated the successful synthesis and thorough characterization of two new isostructural highly porous metal phosphonates of composition [M(Ni-H_2_TPPP)(OH/F)_2_] (M = Zr, Hf) (M-CAU-30) containing a Ni-metalated porphyrin-based phosphonate as linker molecule. Although both compounds are only obtained with ZrO_2_ and HfO_2_ as impurities, respectively, sorption measurements result in high specific surface areas of 970 and 910 m^2^ g^–1^ for the reaction products which corresponds to values of 1080 and 1030 m^2^ g^–1^ for the phase pure materials. Thus they exhibit to the best of our knowledge the highest specific area for porous metal phosphonates ever reported. Furthermore CAU-30 reveals remarkably high thermal and chemical stability of 400 °C and in a pH range between 0 and 12 as well as in phosphate buffer. The redox activity of Zr-CAU-30 was investigated by cyclic voltammetry resulting in a reversible redox process at a half-wave potential of *E*_1/2_ = –0.649 V. CAU-30 stands out due to its thermal and chemical stability which makes CAU-30 a promising candidate for many applications discussed in the context of MOFs, from conventional gas-separation to storage over catalysis or proton conductivity. The fact that a Ni^2+^ ion is connected to the centre of the porphyrin ring opens applications in catalysis due to the catalytic activity of the free Ni metal sites. In addition free –OH groups of the linker reside in the pores, which makes CAU-30 a possible candidate for proton conductivity where stability in a large pH range is required.

## Experimental

### Materials

ZrOCl_2_·8H_2_O (99.5%, Sigma Aldrich), HfCl_4_ (98%, Sigma Aldrich), NiCl_2_·6H_2_O (97%, Merck), EtOH (99% + 1% MEK, Walther), dichloromethane (Walther), *N*,*N*-dimethylformamide (99%, Grüssing), acetone (99%, Walther), 100% acetic acid (VWR chemicals), NaH_2_PO_4_ (99%, Sigma-Aldrich) and Na_2_HPO_4_ (99%, Sigma-Aldrich) were used without further purification. The linker Ni-4,4′,4′′,4′′′-(5,10,15,20-porphyrintetrayl)-tetraphosphonobenzoic acid (Ni-tetra(4-phosphonophenyl)porphyrin, Ni-H_8_TPPP) was synthesized according to reported procedures^67^–^69^ starting with 4-bromobenzaldehyde (99%, Sigma Aldrich) and pyrrole (98%, ABCR) in propionic acid (99%, Grüssing) with following NiCl_2_-catalyzed (97%, Merck) phosphonylation in 1,3-diisopropylbenzene (96%, Sigma-Aldrich) using triethyl phosphite (98%, Sigma-Aldrich) with following hydrolysis in conc. HCl (35%, VWR chemicals). Details are given in the ESI.†

### Preparation

The synthesis of M-CAU-30 (M = Zr, Hf) was studied using high-throughput methods (*V*_max_ = 2.0 mL per Teflon insert) with H_2_O as solvent.^70^ Reaction temperatures between 120 and 180 °C, reaction times between 6 and 48 h and different molar ratios of metal to linker to NaF to NaOH in a range between 1 : 1 : 10 : 1 and 5 : 1 : 120 : 16 were employed (1 equivalent corresponds to 5.3 × 10^–3^ mmol). Various additives like HCl, formic or acetic acid were employed, but only the use of NaF in combination with NaOH resulted in highly porous compounds. The influence of fluoride ions in the synthesis of porous Zr-phosphonates has been previously discussed in the literature.^35^,^41^ Furthermore an upscaling of the synthesis of M-CAU-30 in 30 mL reactors was successfully carried out by using the 12-fold amounts of all reactants. Reactions under stirring using the 3-fold amounts were carried out in 5 mL glass vials. Under these conditions the highly crystalline compounds were already obtained at a reaction temperature of 160 °C within 3 h.

The optimized synthesis conditions for both title compounds in 5 mL glass vials are given in the following paragraphs.

#### Zr-CAU-30

Ni-H_8_TPPP (15.0 mg, 15.9 × 10^–3^ mmol), ZrOCl_2_·8H_2_O (9.8 mg, 33.0 × 10^–3^ mmol), NaF (38.2 mg, 9.6 × 10^–1^ mmol), H_2_O (3000 μL), 2 M aqueous solution NaOH (60.6 μL, 12.9 × 10^–2^ mmol) were added to a 5 mL glass vial and placed in a reaction block on a heating plate. The block was heated for 3 h at 160 °C under stirring. The resulting product was centrifuged at 6000 rpm for 10 min and washed once with H_2_O and acetone each. A yield of 6.7 mg was obtained which consist of a mixture of ZrO_2_ and Zr-CAU-30, [Zr_2_(Ni-H_2_TPPP)(OH/F)_2_]·23H_2_O.

Elemental analysis of Zr-CAU-30 [Zr_2_(Ni-H_2_TPPP)(OH/F)_2_]·23H_2_O: calc. (%): C 30.1, H 5.9, N 3.2, found (%): C 28.5, H 3.3, N 3.0. Elemental analysis of a one to one mixture of ZrO_2_ and [Zr_2_(Ni-H_2_TPPP)(OH/F)_2_]·23H_2_O: calc. (%): C 28.9, H 5.0, N 3.1, found (%): C 28.5, H 3.3, N 3.0.

#### Hf-CAU-30

Ni-H_8_TPPP (15.0 mg, 15.9 × 10^–3^ mmol), HfCl_4_ (9.7 mg, 3.3 × 10^–2^ mmol), NaF (38.2 mg, 9.6 × 10^–1^ mmol), H_2_O (3000 μL), 2 M aqueous solution NaOH (60.6 μL, 12.9 × 10^–2^ mmol) were added to a 5 mL glass vial and placed in a reaction block on a heating plate. The block was heated for 3 h at 160 °C under stirring. The resulting product was centrifuged at 6000 rpm for 10 min and washed once with H_2_O and acetone each. A yield of 7.0 mg was obtained which consist of a mixture of HfO_2_ and Hf-CAU-30, [Hf_2_(Ni-H_2_TPPP)(OH/F)_2_]·30H_2_O.

Elemental analysis of Hf-CAU-30 [Hf_2_(Ni-H_2_TPPP)(OH/F)_2_]·30H_2_O: calc. (%): C 29.0, H 5.0, N 3.1, found (%): C 24.9, H 3.5, N 2.6.

Elemental analysis of a one to one mixture of HfO_2_ and [Hf_2_(Ni-H_2_TPPP)(OH/F)_2_]·30H_2_O: calc. (%): C 24.3, H 4.2, N 2.6, found (%): C 24.9, H 3.5, N 2.6.

To activate the samples and to remove X-ray amorphous side-products as observed in form of Na^+^ impurities in the EDX analysis the samples were stirred for 24 h in 0.1 M aqueous HCl solution, followed by washing with acetone and activation under reduced pressure of 10^–2^ kPa at 170 °C for 16 h.

### Characterization

The crystal structure of Zr-CAU-30 was investigated by combining electron diffraction tomography with powder diffraction data.

For the TEM experiments (TEM, STEM, ADT, HRTEM), a small quantity of Zr-CAU-30-as was dispersed in ethanol using an ultrasonic bath. The dispersion was transferred into a caved tip with a pipette and then sprayed onto standard 300 mesh Cu TEM grids with a thin amorphous carbon film, using an ultrasonic vaporizer. Phase contrast TEM, scanning TEM (STEM), and automated diffraction tomography (ADT) measurements were carried out using a TECNAI F30 S-TWIN transmission electron microscope equipped with a field emission gun and operating at 300 kV. STEM images were collected using a Fischione high-angle annular dark field (HAADF) detector. TEM images and nano electron diffraction (NED) patterns were acquired with a 4k × 4k Gatan US4000 CCD camera (Gatan, Pleasanton, USA). In order to increase the stability of the sample under the electron beam, it was cooled down to about 97 K using a cryo-transfer holder filled with liquid N_2_ after insertion into TEM. Electron diffraction data were collected with an automated acquisition module developed for FEI microscopes.^14^ A Gatan cryotransfer tomography holder (model 914) with a tilt range of ±70° was used for electron diffraction data acquisition. A small condenser aperture of 10 μm, weak gun lens and large condenser spot size were used in order to reduce the electron dose rate on the sample. The crystal position was tracked in microprobe STEM mode and electron diffraction patterns were collected using the above settings. The beam size was set to 100 nm in diameter. In order to integrate reflection intensities over the full tilt wedge, ADT was coupled with precession electron diffraction (PED),^71^,^72^ which was performed using a NanoMEGAS DigStar unit. The precession angle of the beam was kept at 1°. ADT tilt series were collected sequentially in a fixed tilt step of 1°. The exposure time for each frame was set to 3 seconds. TEM in-line holography^73^ was realized by focal series reconstruction from image series collected using a TECNAI F30 ST operating at 300 kV, without aberration corrector, under suitable TEM conditions. 20 images were recorded at a primary magnification of 390 000 with 10 nm focal increment, thus covering a focal range of 190 nm including Gaussian focus. The accumulated dose per focal series was about 90 e^–^ Å^–2^. The images were hardware-binned by 2 resulting in 2k × 2k images with a physical pixel size of 0.057 nm. After image alignment, a 320 × 320 pixel region was chosen for exit wave reconstruction, employing a Gerchberg–Saxton algorithm programmed in Python. Residual axial aberrations were corrected by an automated minimization routine implemented in Python.

Further TEM investigations, standard ED and energy dispersive X-ray (EDX) spectroscopy were performed on a Tecnai G2 20 S-TWIN (FEI) at 200 kV and on a Titan Themis 60-300 (FEI) equipped with a SuperX EDX detector, operated at 300 kV.

Crystal structure solution of Zr-CAU-30-as was carried out from ADT data. The ADT3D software^15^,^72^ was used for processing the three-dimensional electron diffraction data yielding unit–cell parameters, symmetry information and reflection intensities. The unit–cell parameters were refined with a Pawley fit against PXRD data. The *ab initio* structure solution using direct methods as implemented in SIR2014 (ref. 74) was based on the reflection intensities derived from electron diffraction data.

IR spectra were recorded using a Bruker ALPHA-FT-IR A220/D-01 spectrometer equipped with an ATR-unit. Additional FTIR spectra of an *in situ* activated sample of Zr-CAU-30 were recorded on a Thermo-Fischer NICOLET 6700 spectrometer featuring a DTGS detector. The material was pressed into both a self-supporting pellet and a KBr pellet containing 2.5 wt% Zr-CAU-30. Prior to measurements, adsorbed water was removed *in situ* by heating the pellets at 200 °C for 2 h under vacuum. Spectra of 128 scans were recorded at 25 °C in the 500–4000 cm^–1^ range, with a 2 cm^–1^ resolution. UV/vis spectra were recorded on a Spectroquant Pharo 300 M spectrometer. For the measurement 0.1 mg of the respective sample was dissolved in 1 mL 2 M aqueous NaOH solution and diluted with water to a total volume of 5 mL. Afterwards a 4 mL cuvette was filled with 3 mL water and 120 μL of the porphyrin containing solution was added to the cuvette and subsequently measured. The ^1^H- and ^31^P-NMR spectra of the Ni-H_8_TPPP linker were recorded on a Bruker DRX 500 spectrometer after digestion in DMSO-d_6_. Sorption experiments were performed with a BEL Japan Inc. BELSORP-max. Before sorption measurements all samples were activated at 170 °C under reduced pressure (10^–2^ kPa) for 16 h. Thermogravimetric measurements were performed on a NETZSCH STA 409 CD analyser under a flow of air (75 mL min^–1^) with a heating rate of 4 °C min^–1^ between 25 and 1000 °C in Al_2_O_3_ crucibles. The data were corrected for buoyancy and current effects. Cyclic voltammograms (CV) were measured on a EG&G Princeton Applied Research/Model 273A with Ag/AgCl 207 mV *vs.* NHE type reference electrode and Pt as counter electrode. As electrolyte 0.1 M phosphate buffer (pH 7) and as working electrode FTO (fluorine doped tin oxide) on glass (Sigma) 100 nm thickness, surface resistivity 7 ohm per cm^2^ was employed. For the preparation of the FTO electrode 5 mg Zr-CAU-30, 30 μL Nafion (5% in lower aliphatic alcohols and water) and 143 μL EtOH were added to 857 μL H_2_O followed by ultrasonication for 30 min. The mixture was dropcasted on a 1 cm^2^ area of the FTO electrode and dried overnight under atmospheric conditions.

### Structure refinement

The PXRD pattern of the activated compound matches well with a tetragonal unit cell related to the one observed by electron diffraction methods. In comparison to the PXRD pattern of Zr-CAU-30-as additional reflections are observed in the PXRD pattern of Zr-CAU-30-act which indicates a change of symmetry upon thermal treatment. The detailed analysis of the extinction conditions indicate the space group *I*4_1_*cd* (no. 110) for CAU-30-act with a lower symmetry than observed for Zr-CAU-30-as (*I*4_1_/*acd* no. 142) from electron diffraction data. Starting with the crystal structure of the framework as determined from electron diffraction data, we set up a model by first converting the space group symmetry from *I*4_1_/*acd* to *I*4_1_*cd* using Powdercell^58^ and subsequent optimisation of the structure using the universal force field as implemented in Materials Studio.^59^ While the small number of reflections in combination with the very large unit cell prevented a full refinement, the crystal structure could still be approximated by Rietveld methods using TOPAS.^75^ Hence, the position of the porphyrin moiety was kept fixed and the phenylphosphonate fragments were treated as rigid bodies while the remaining zirconium and oxygen atom were only refined along their *x* and *y* coordinates. Moreover the intensities can be only fitted when considering a preferred orientation along (110) and taking into account some residual electron density, represented by partially occupied oxygen atoms (designated as G*n*). The electron density of G1 is positioned in chemically unreasonable distance to the linker molecules (1.7(3) Å) and therefore we assume that this rather indicates further positional disorder of the linker molecules in the crystal structure. However, further modelling of this disorder is beyond the capability of the method, also taking into account the quality of the PXRD data. Residual electron density in the pores were refined as oxygen atoms of water molecules. This fact indicates that a complete removal of all solvent molecules is not possible by activation at elevated temperatures in vacuum. Some relevant final parameters and the final plot for the Rietveld-based approximation are given in Table 1 and Fig. S7b.†

High resolution PXRD patterns were measured on a STOE Stadi-P combi powder diffractometer equipped with a Mythen detector (Cu Kα_1_ radiation). Pawley fits (Fig. S7a, c†) were carried out for M-CAU-30 (M = Zr, Hf) to confirm phase purity. Pawley fits were performed with the program TOPAS Academic V4.1.^75^ The results of the refinements and crystallographic data of M-CAU-30 are shown in Table 1. The diameters of all pores were determined using DIAMOND V.3 (ref. 76) taking the van der Waals radii of the atoms into account. The variable temperature (VT) PXRD measurements were recorded on a STOE Stadi-P combi powder diffractometer (Mo Kα_1_ radiation) equipped with a capillary furnace. For the measurements 0.5 mm quartz capillaries were used. The samples were heated up in steps of 5 K between 250 and 450 °C and measured for 10 minutes each.

## Conflicts of interest

There are no conflicts to declare.

## Supplementary Material

Supplementary information

Crystal structure data
