# Supplementary material for: Highly stable and porous porphyrin-based zirconium and hafnium phosphonates – electron crystallography as an important tool for structure elucidation
Source: Chem Sci. 2018 May 28;9(24):5467–78. doi: 10.1039/c8sc01533c (PMC6009505; doi:10.1039/c8sc01533c)
Supplement: Supplementary file 1 [file SC-009-C8SC01533C-s001.pdf]

Electronic Supplementary Material (ESI) for Chemical Science.
This journal is © The Royal Society of Chemistry 2018

Supporting Information

Highly stable and porous porphyrin-based zirconium and hafnium phosphonates - Electron crystallography as an important tool for structure elucidation

Timo Rhauderwiek,[a] Haishuang Zhao,[b] Patrick Hirschle,[c] Markus Döblinger[c], Bart Bueken[d], Helge Reinsch,[a] Dirk de Vos,[d] Stefan Wuttke,[c] Ute Kolb[b] and Norbert Stock[a]

[a] Institute of Inorganic Chemistry, Christian-Albrechts-Universität, Max-Eyth Straße 2, D-24118 Kiel, Germany.

[b] Institute of Inorganic Chemistry and Analytical Chemistry, Johannes Gutenberg-University Mainz, Jakob-Welder-Weg 11, D-55128 Mainz, Germany

[c] Department of Chemistry and Center for NanoScience (CeNS), University of Munich (LMU), Butenandtstraße 5-13, D-81377 Munich, Germany.

[d] Centre for Surface Chemistry and Catalysis, KU Leuven, Celestijnenlaan 200f, B-3001 Leuven, Belgium.

1. **^{1}H-NMR spectroscopy of Ni-H_8TPPP, list of known Zr-phosphonates and high-throughput investigations……...2-7**
2. **Electron diffraction tomography and Pawley-/ Rietveld-refinements...…………..8-13**
3. **H_2O Sorption properties and PXRD patterns of the activated samples……..………14**
4. **TG investigations, PXRD patterns of the decomposition products, VT-PXRD studies and chemical stability………………………………………………………………15-18**
5. **Spectroscopy ……………………………………………………………...19-20**
6. **TEM and SEM measurements………………………………………………..21-27**
7. **Cyclic voltammetry………………………………………………………………..28**
8. **References……………………………………………………………………………29**

1. ^{1}H-NMR spectroscopy of Ni-H_8TPPP, list of known Zr-phosphonates and high-throughput investigations

Synthesis of Ni-H_8TPPP. The three step synthesis of Ni-H_8TPPP (Fig. S1) was carried out employing in the first step the Adler method[7, 8] for the synthesis of 4-Bromophenylporphyrin (H_2TBrPP). The second step, the phosphorylation of H_2TBrPP was achieved by using a Ni-catalyzed variation of the Arbuzov reaction,[9, 10] with the following third step, the hydrolysis of the -$PO(OEt)_2$ groups in conc. HCl.

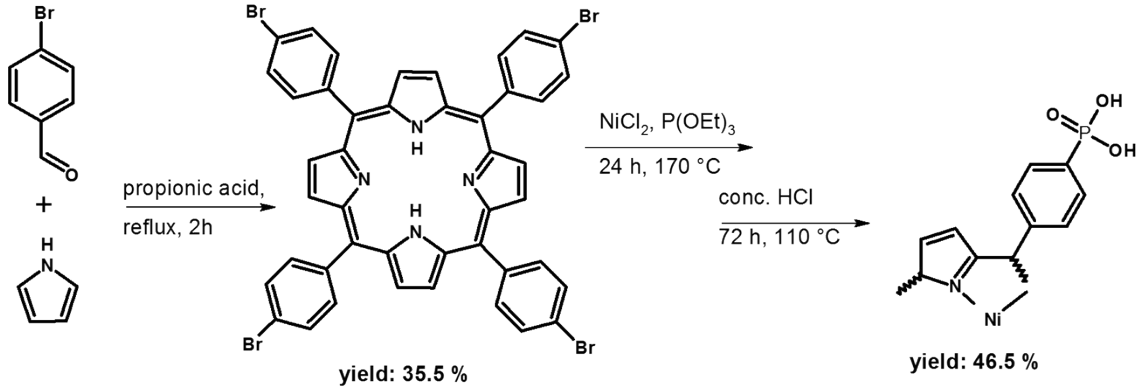

Figure S1. Schematic representation of the synthesis of Ni-H_8TPPP.

H_2TBrPP: Pyrrole (7.47 mL, 108 mmol) and 4-brombenzaldehyde (20.0 g, 108 mmol) in 400 mL propionic acid were heated under reflux for 2 h. Afterwards the reaction mixture was cooled down to room temperature and poured into 500 mL of MeOH and stirred for 30 min in an ice bath. The resulting precipitate was filtered off and washed several times with MeOH until the filtrate was only slightly red. Subsequently, the product was washed with 100 mL warm distilled water and dried at 70 °C in a drying oven over night. A blue powder of H_2TBrPP 7.63 g (8.2 mmol, 30.4 %) was obtained.

Elemental analysis of H_2TBrPP ($Br_4C_{44}H_{26}N_4$): calc (%): C 56.8, H 2.6, N 6.0, found (%): C 59.7, H 3.2, N 6.2. The small difference between the calculated and observed values is due to impurities caused by solvent molecules used in the synthesis. These impurities were also observed in the ^{1}H-NMR measurements.

^{1}H-NMR (Fig. S2), H_2TBrPP (500 MHz $CDCl_3$): δ= 8.76 (s, 8H, H-1); 7.99 (d, 8H, H-2); 7.82 (d, 8H, H-3); 7.18 (s, 1H, $CDCl_3$); 2.30 (q, 2H, propionic acid); 1.08 (t, 3H, propionic acid) ppm. Additional peaks between 7 and 9 ppm are due to small impurities with porphyrinic oligomers.

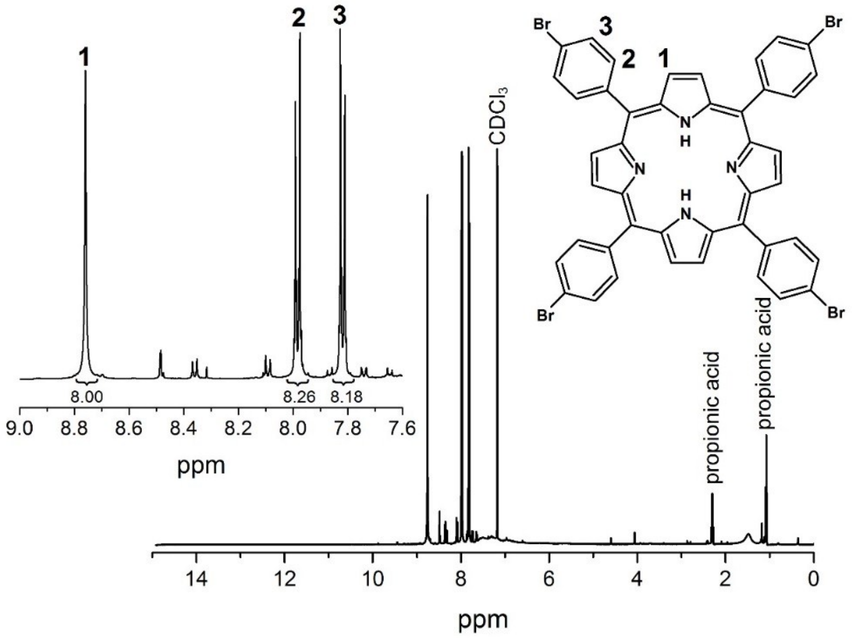

Figure S2. ^{1}H-NMR spectrum of H_2TBrPP digested in DMSO.

Ni-H_8TPPP: H_2TBrPP (3.42 g, 3.7 mmol) and anhydrous $NiCl_2$ (1.79 g, 13.8 mmol) in 70 mL 1,3-diisopropylbenzene (prior the reaction for 30 min with N_2 degassed) were heated up to exactly 170 °C under N_2 atmosphere. Afterwards triethylphosphite (7.05 mL, 43.7 mmol) was added slowly (within 30 min) and dropwise to the reaction mixture and heated for further 24 h at 170 °C. The reaction mixture was cooled down to room temperature and the resulting precipitate was filtered off and washed with 250 mL CH_2Cl_2. The resulting organic phase was washed 2 times each with 100 mL H_2O. The organic solvents were evaporated under reduced pressure and the precipitate was hydrolyzed with 80 mL conc. HCl at 110 °C in 72 h. The resulting product was filtered off, washed with 100 mL cold water and afterwards stirred in 100 mL CH_2Cl_2 for 24 h. The clean product was filtered off and dried at 70 °C in a drying oven over night. A red powder of Ni-H_8TPPP 2.74 g (2.76 mmol, 74.6 %) was obtained.

Elemental analysis of Ni-H_8TPPP ($C_{44}H_{32}N_4NiO_{12}$) · $4H_2O$: calc (%): C 49.8, H 3.6, N 5.3, found (%): C 49.1, H 3.6, N 5.1.

^{1}H-NMR (Fig. S3), Ni-H_8TPPP (500 MHz DMSO-d_6): δ= 8.74 (s, 8H, H-1); 8.11 (dd, 8H, H-2); 8.06 (dd, 8H, H-3); 2.50 (s, 6H, DMSO-d_6); 2.09 (s, 6H, acetone) ppm.

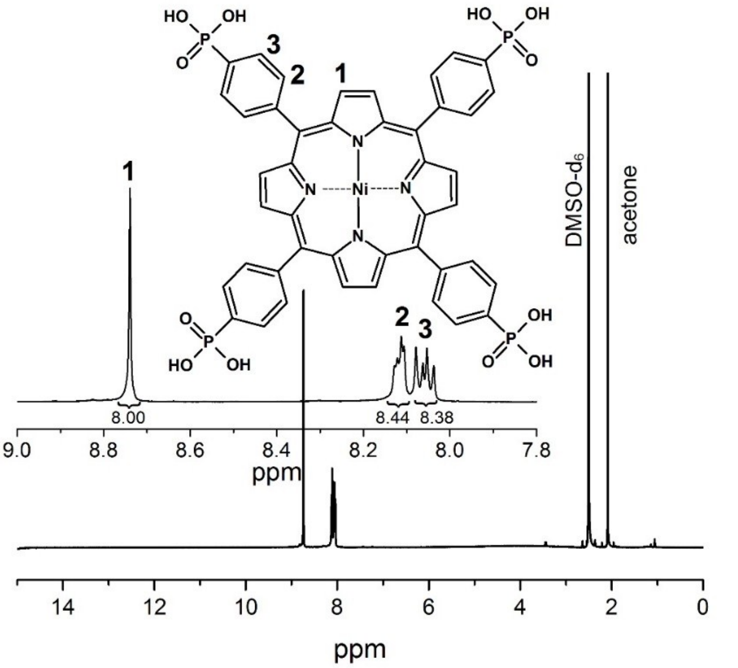

Figure S3. ^{1}H-NMR spectrum of Ni-H_8TPPP digested in DMSO.

^{31}P-NMR (Fig. S4), Ni-H_8TPPP (500 MHz DMSO-d_6): δ= 12.04 (s, 4P, -PO(OH)$_2$) ppm.

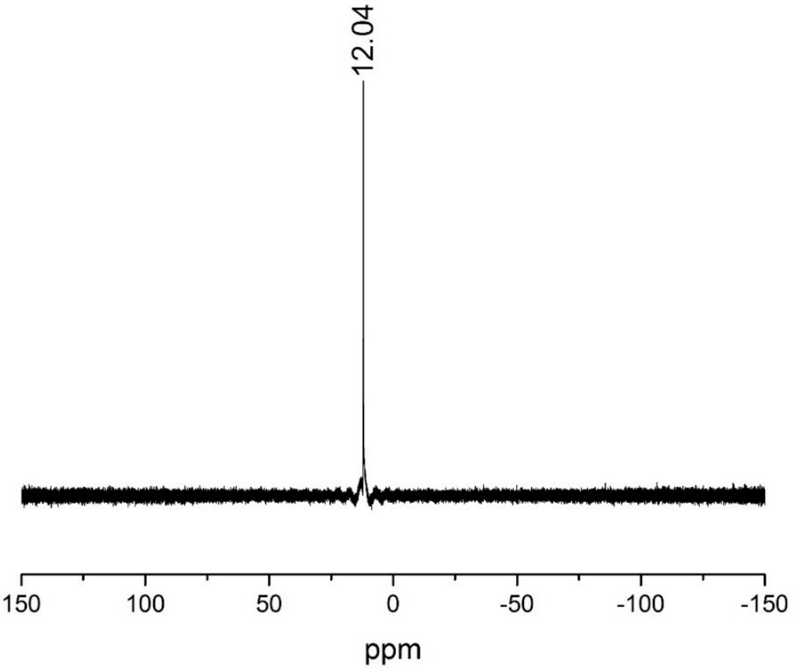

Figure S4. ^{31}P-NMR spectrum of Ni-H_8TPPP digested in DMSO.

Table S1. Overview of porous and crystalline Zr-phosphonates. In addition to the names the formula, the specific surface areas and the space group symmetry of the crystal structure are given.

name[Lit.]	formula	Specific surface area	SG
CALF-31[4]	$[Zr(H_2PPB)]$<br>H_6PPB= 1,3,5-*Tris*-(4-phosphonophenyl)benzene	793 m^2 g^{-1}	model
ZrPPB[3]	$[Zr_3(H_3PPB)_4]$	10 m^2 g^{-1}	$P\bar{3}1c$
ZrPBMP[1]	$[Zr_2H_4(PBMP)_3]$<br>H_4PBMP= N,N'-piperazin *Bis*methylenphosphonic acid	-	$R\bar{3}$
UPG-1[2]	$[Zr(H_4PMPT)_2]$<br>H_6PMPT= 2,4,6-Tris(4-(phosphonomethyl) phenyl)-1,3,5-triazine	410 m^2 g^{-1}	$R\bar{3}$
SZ-1[6]	$[C_4mpyr][Zr_{2.5}(H_3TPPM)F_6]$<br>$C_4mpyr^+$= N-butyl-N-methylpyrrolidinium<br>H_8TPPM=Tetrakis(4-phosphonophenyl)methane	10 m^2 g^{-1}	*Pbca*
SZ-2[6]	$[C_4mpyr]_2[Zr_{1.5}(TPPM)_{0.5}F_4]$	225 m^2 g^{-1}	*I4/m*
SZ-3[6]	$[C_2py]_2[Zr_{3.5}(HTPPA)F_9]$<br>$C_2py^+$=N-ethylpyridinium<br>$H_8TPPA$= 1,3,5,7-Tetrakis(4-phosphonophenyl) adamantane	594 m^2 g^{-1}	$P2_1/n$

High-throughput investigations

High-throughput reactors (2 mL Teflon inserts) were employed in the screening of synthesis parameters such as molar ratio of metal to linker to NaF to NaOH, pH and reaction temperature and time (Fig. S5a). The use of 0 and 12 eq. NaOH resulted in X-ray amorphous products while 4 and 8 eq. NaOH resulted in CAU-30 (1 eq. corresponds to 5 mg Ni-H_8TPPP ($5.3 \cdot 10^{-3}$ mmol, Fig S5b). A molar linker to metal ratio of 1 : 1 resulted in CAU-30 with low crystallinity, while a ratio of ≥ 1 : 2 led to highly crystalline CAU-30. Furthermore an upscaling of CAU-30 in 30 mL Teflon inserts was possible using the 12 fold amount of all reactants and solvents. By using 5 mL glass vials and stirring of the reaction mixture, the reaction temperature could be reduced from 48 to 3 h and the reaction temperature from 180 to 160 °C (Fig. S5b)

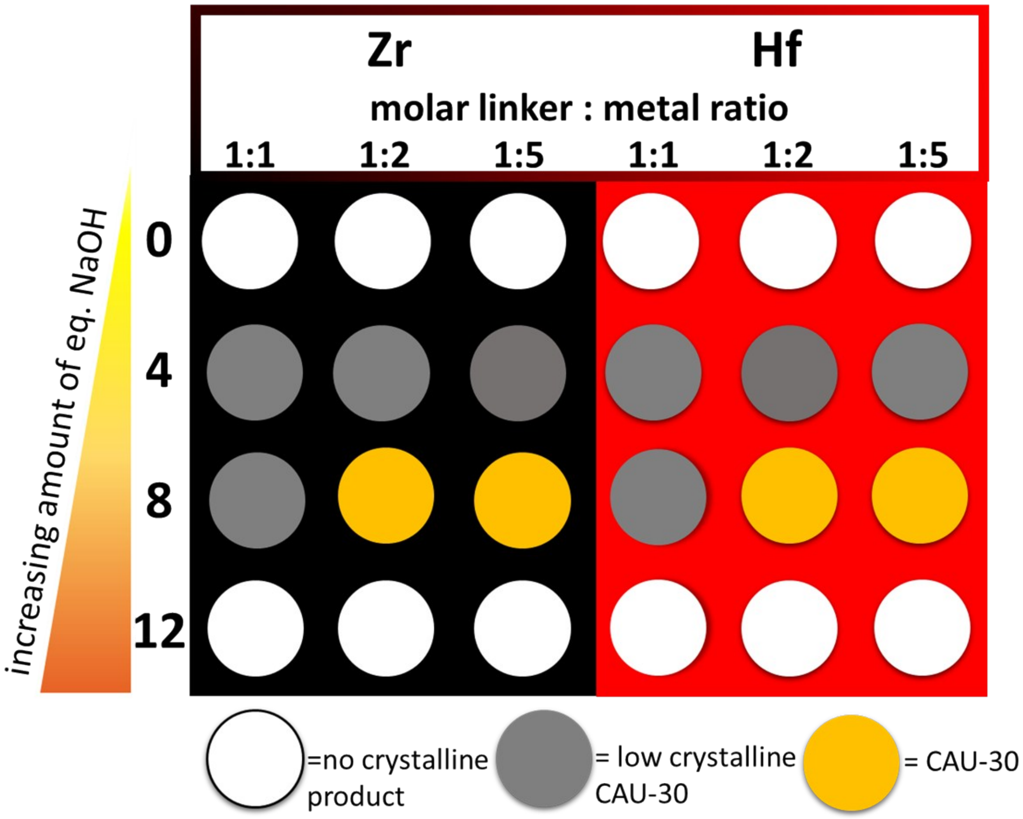

Figure S5a. Results of the high-throughput investigation of M-CAU-30 (M= Zr, Hf) varying the molar ratio linker to metal to NaOH. In all reactions 60 eq. of NaF (1 eq. corresponds to 5 mg Ni-H_8TPPP ($5.3 \cdot 10^{-3}$ mmol)) were used.

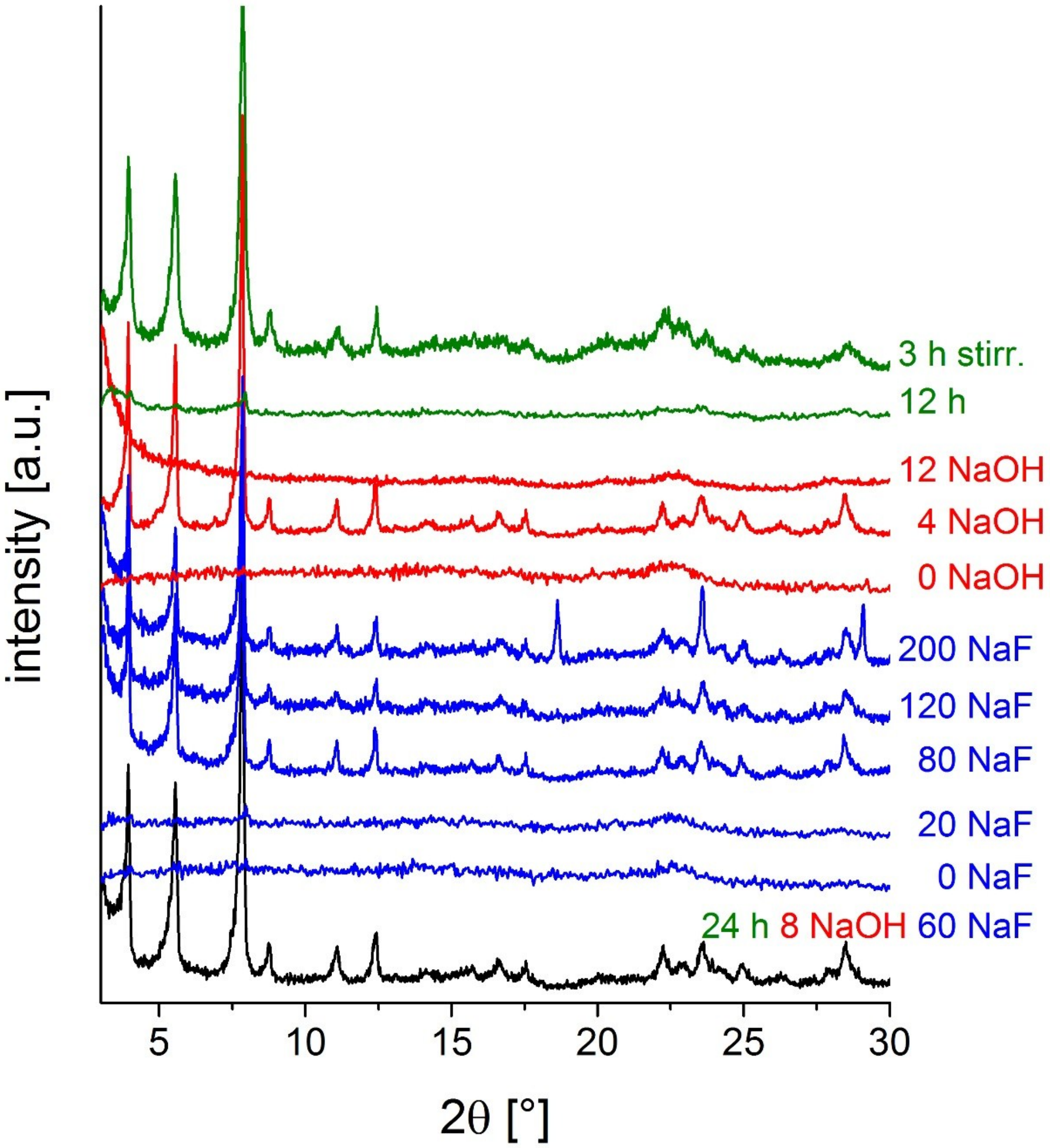

Figure S5b. PXRD patterns obtained from the reaction products of the high-throughput investigation of M-CAU-30 (M= Zr, Hf). The black pattern corresponds to the product obtained using the final optimized synthesis conditions (24 h without stirring, 8 eq. NaOH, 60 eq. NaF). The blue and the red patterns demonstrate the influence of the amount of NaF (24 h, 8 eq. NaOH) and NaOH (24 h, 60 eq. NaF), respectively, on the product formation. The influence of stirring and changing the reactor from a Teflon reactor to a glass vials is shown in the green PXRD patterns: the reduction of the reaction time (8 eq. NaOH, 60 eq. NaF) to 12 h without stirring and 3 h under stirring results in an X-ray amorphous and a crystalline product, respectively. A value of 1 eq. corresponds $5.3 \cdot 10^{-3}$ mmol.

2. Electron diffraction tomography and Pawley / Rietveld refinements.

STEM images and 2D slices of the reciprocal lattice of Zr-CAU-30 are given in Figures S6a to S6d.
The crystallographic data of M-CAU-30 (M= Zr, Hf) obtained by Pawley- and Rietveld-refinements reveal very similar unit cell parameters and the resulting Rietveld- and Pawley-refinements prove that the two CAU-30 derivatives are isostructural and contain no further crystalline impurities (Fig. S7a – S7c, Tab.S2).

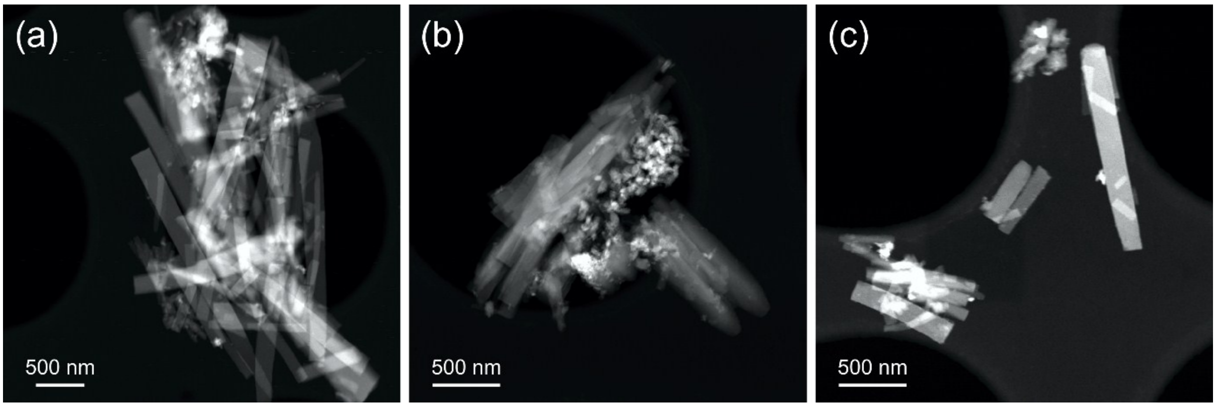

Figure S6a: STEM images of Zr-CAU-30: (a, b) overview of nanorods; (c) single separate nanorods.

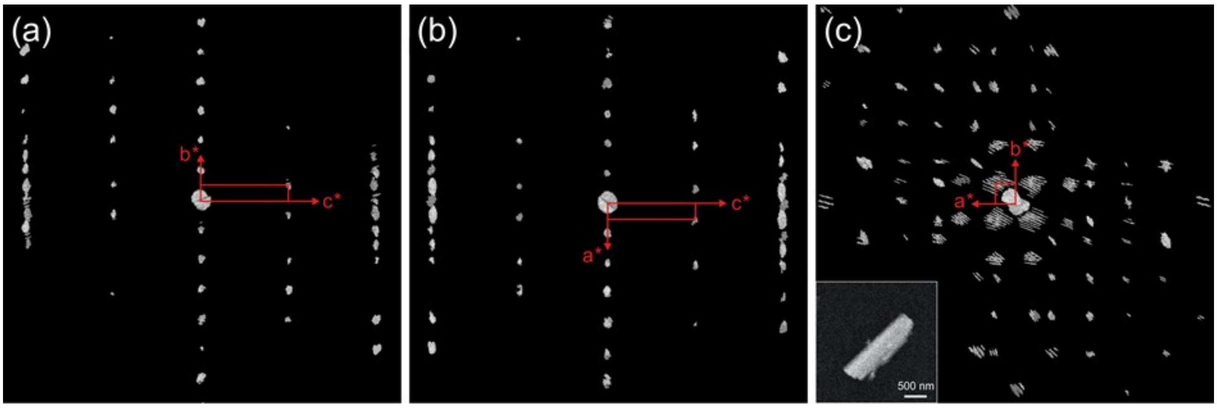

Figure S6b. Reconstructed three-dimensional reciprocal space obtained from ADT data viewed down the three main axes (*a* - *c*), delivering *a* = 46.09 Å, *b* = 45.74 Å, *c* = 7.81 Å, α = 89.0°, β = 90.5°, γ = 91.1°. The inset shows the STEM image of the selected crystal for acquisition of ADT data. The electron beam for data collection was shifted on the crystal at the ADT data acquisition to minimize beam damage.

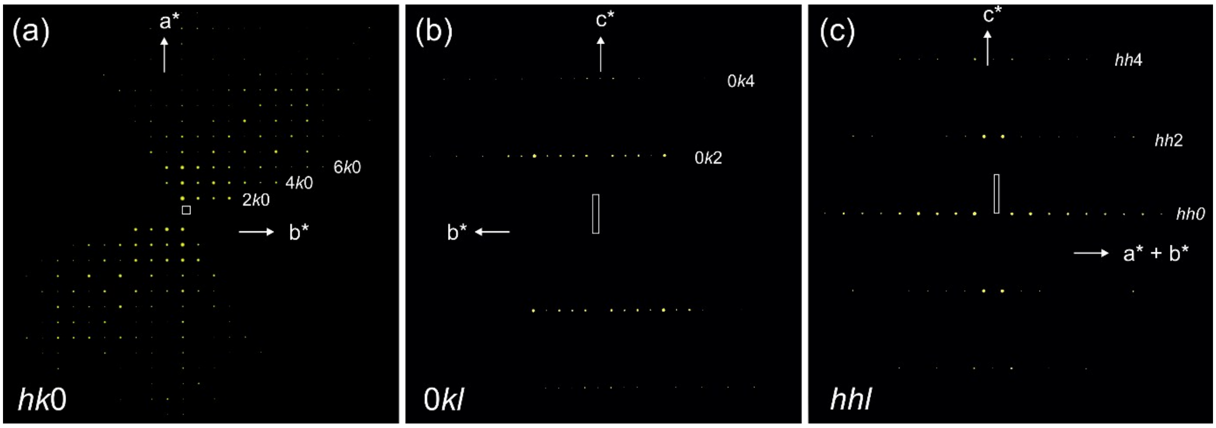

Figure S6c: Three 2D slices *hk*0 (a), 0*kl* (b) and *hhl* (c) cut from reconstructed 3D reciprocal lattice of Zr-CAU-30.

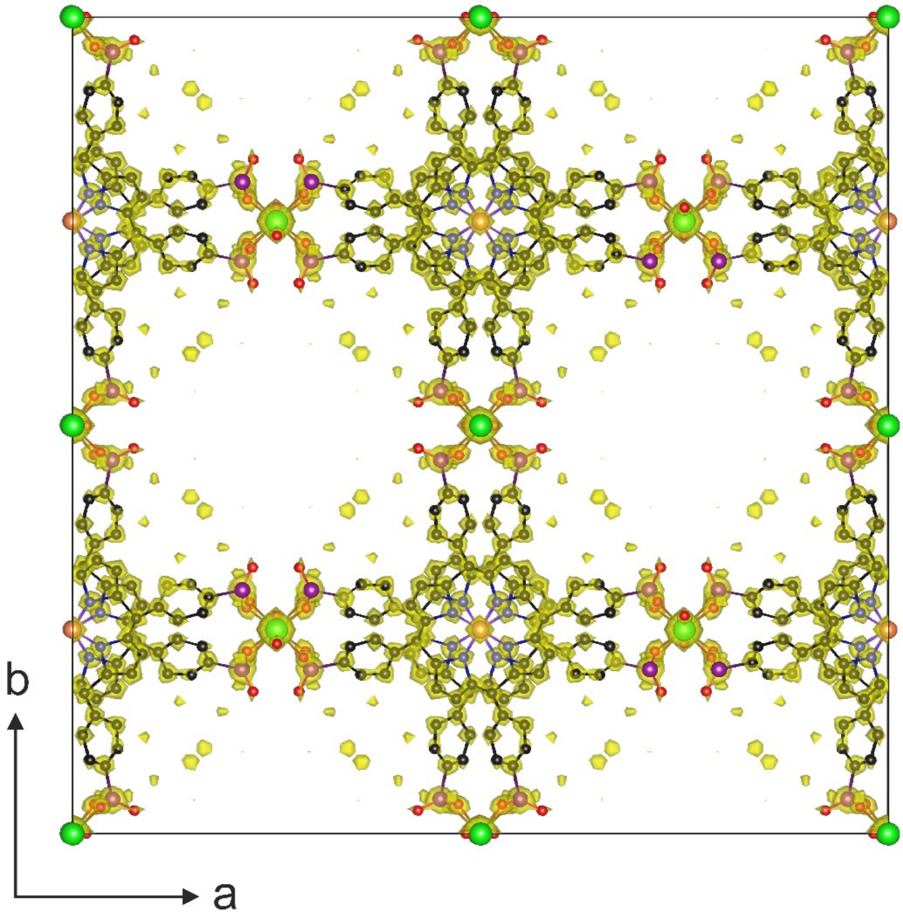

Figure S6d. Fourier potential map of Zr-CAU-30-as derived from the structure solution using ADT data and overlaid atomic framework model (view along [001]). Atom color: green for Zr, yellow for Ni, violet for P, red for O, black for C, blue for N.

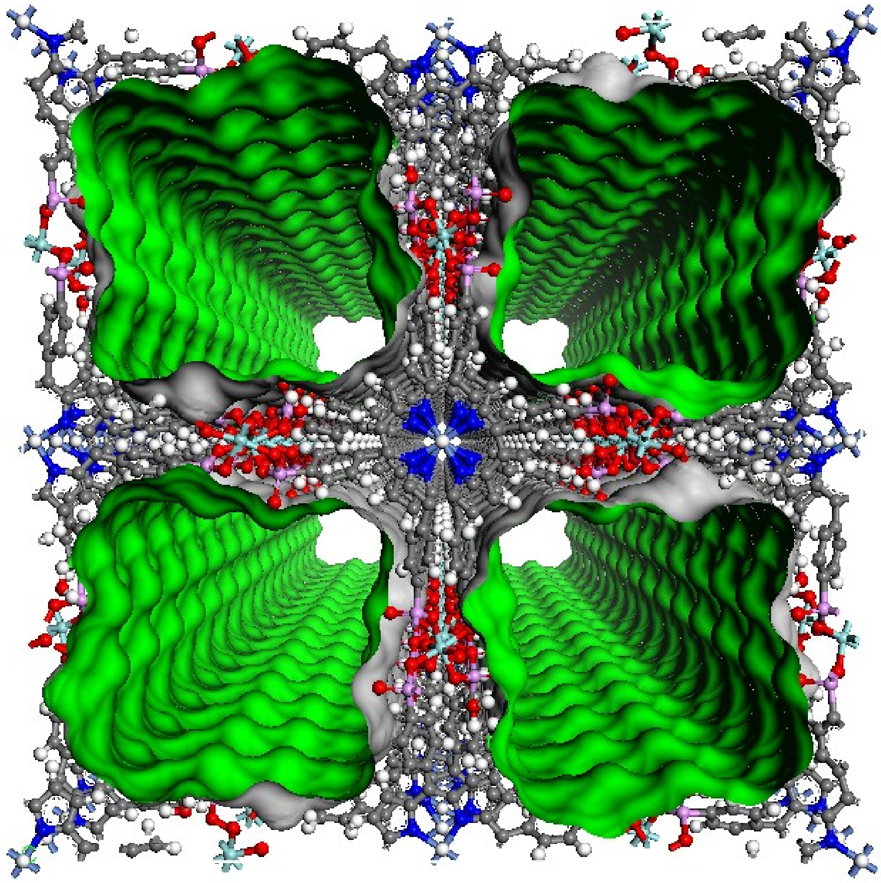

Figure S6e. Connolly Surface of the pores in Zr-CAU-30 along [001] (green, probe radius 1.8 Å).

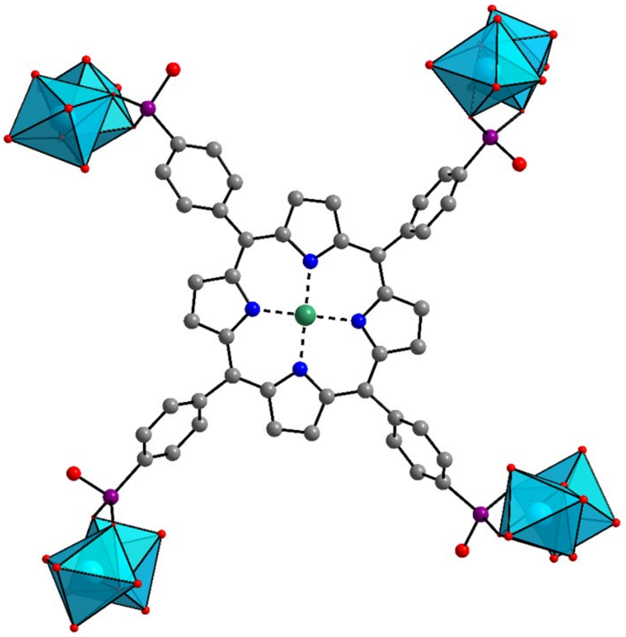

Figure S6f. Coordination mode of the linker molecule. Each linker molecule is coordinated to eight Zr^{4+} ions. Zirconium and ZrO_6 polyhedra are represented in light blue, nickel in green phosphorous in purple, oxygen in red, nitrogen in blue and carbon in grey.

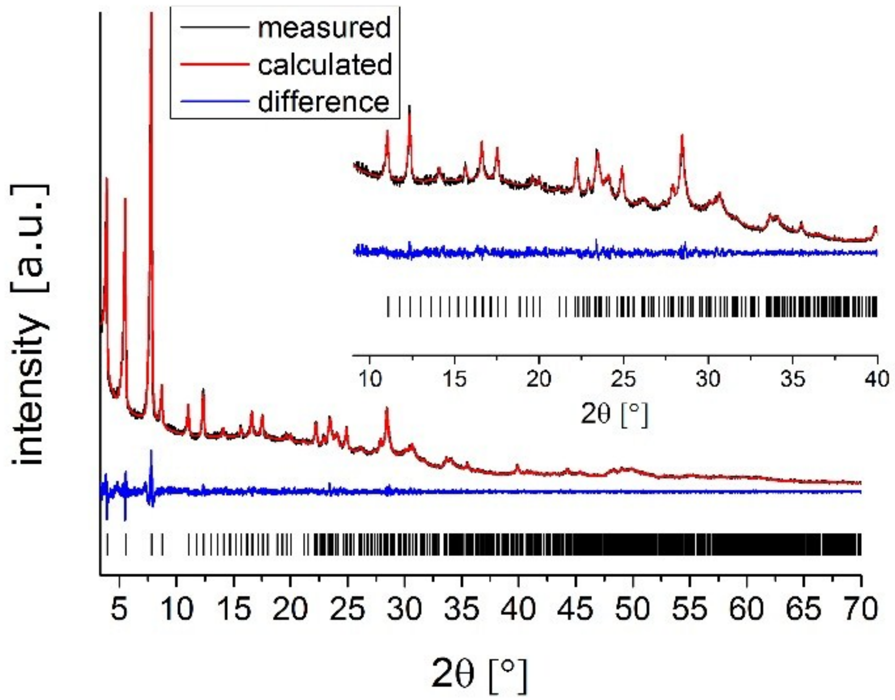

Figure S7a. Results of the Pawley-refinement of Zr-CAU-30-as.

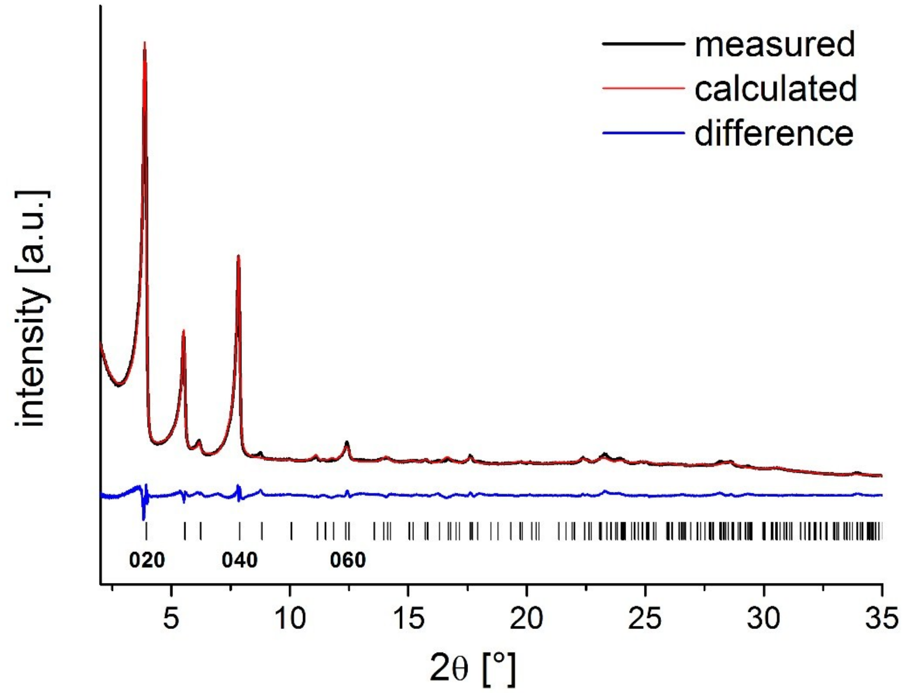

Figure S7b. Results of the Rietveld refinement of Zr-CAU-30-act (250 °C, reduced pressure of 10^{-2} kPa) with mentioned reflections hkl as observed by TEM measurements.

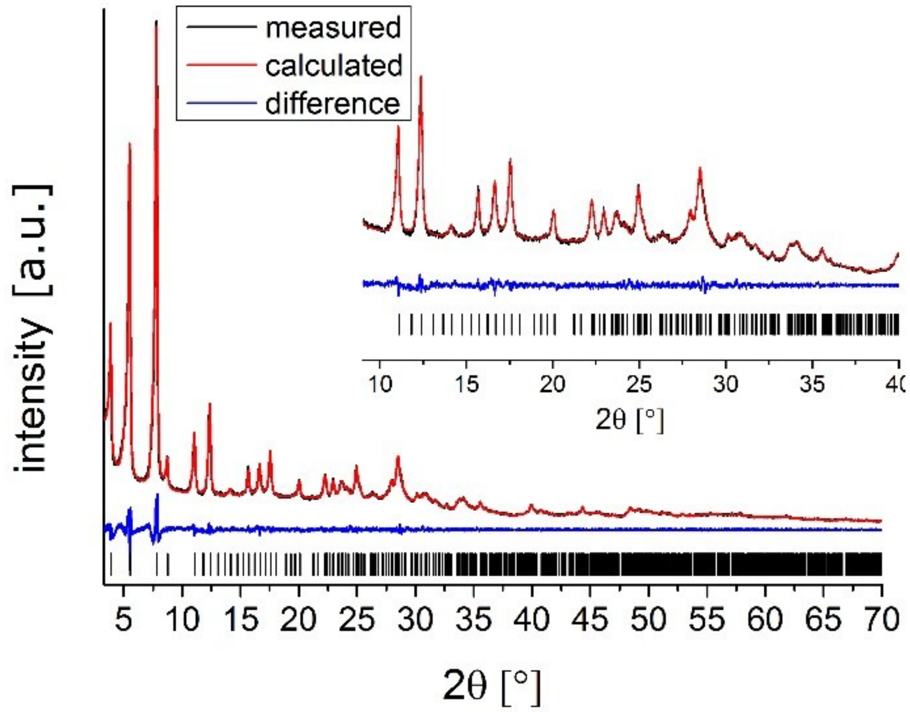

Figure S7c. Results of the Pawley-refinement of Hf-CAU-30-as.

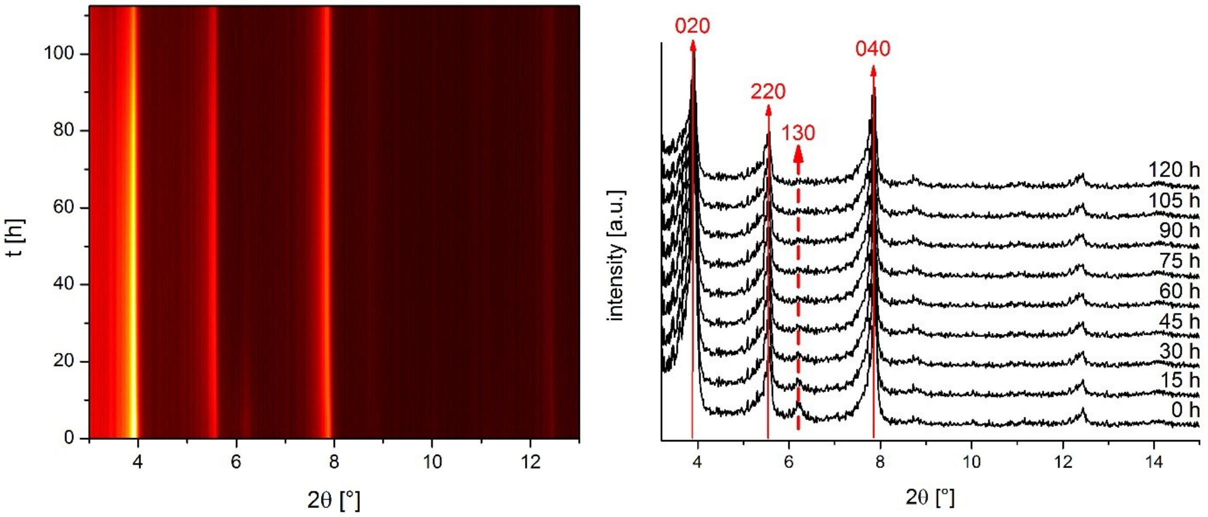

Figure S7d. PXRD patterns of Zr-CAU-30 measured every 5 minutes over 5 days to show the reversibility of the transformation from Zr-CAU-30-act ($I4_1cd$) into Zr-CAU-30-as ($I4_1/acd$), indicated by the disappearance of the 130 reflection at 6.2 ° 2 theta and changes in the relative intensities. Contour-plot (left) and selected PXRD pattern between 0 and 120 h (right).

Table S2. Results of the Rietveld refinement of Zr-CAU-30-act (activated at 250 °C for 2h under reduced pressure of 10^{-2} kPa) and the Pawley refinement of M-CAU-30-as (M= Zr, Hf, as synthesized).

	**Zr-act**	**Zr-as**	**Hf-as**
**determination method**	Rietveld	Pawley	Pawley
**crystal system**	tetragonal	tetragonal	tetragonal
***a* [Å]**	44.778(6)	45.121(5)	45.040(5)
***b* [Å]**	44.778(6)	45.121(5)	45.040(5)
***c* [Å]**	7.658(4)	8.090(2)	8.049(3)
**α [°]**	90	90	90
**β [°]**	90	90	90
**γ [°]**	90	90	90
**V [Å^3]**	15354(8)	16470(5)	16329(8)
**space group**	$I4_1cd$	$I4_1/acd$	$I4_1/acd$
**GoF**	2.0	0.72	0.94
**R_{wp} [%]**	3.8	3.3	3.8

3. H_2O Sorption properties and PXRD patterns of the activated samples.

In the following figures the H_2O sorption isotherms of M-CAU-30 (M= Zr, Hf) (Figure S8) and the PXRD patterns of the samples before and after the sorption experiments are shown (Figure S9).

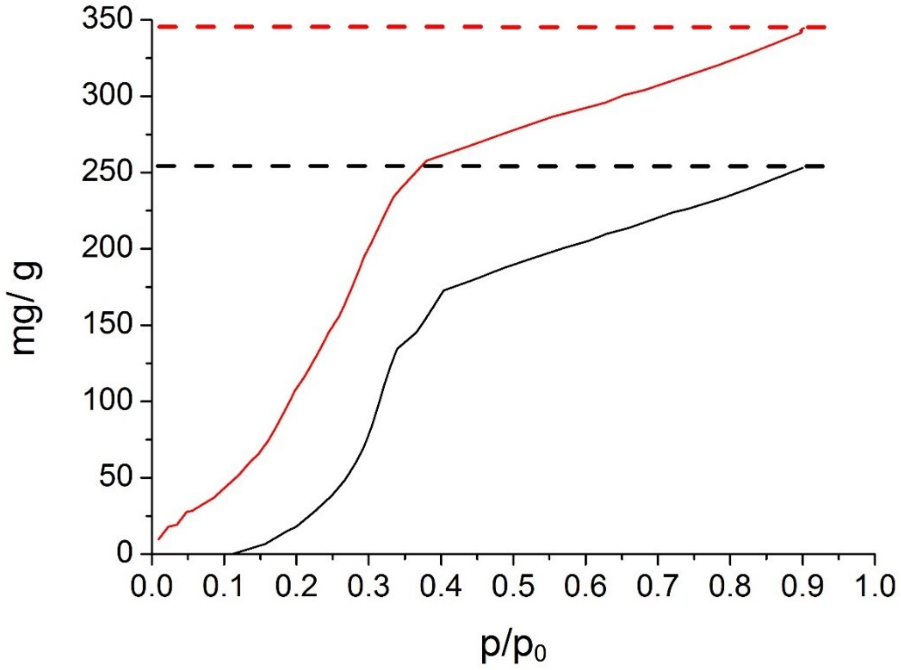

Figure S8. H_2O-sorption isotherms of M-CAU-30 (M = Zr (black), Hf (red)) measured at 298 K.

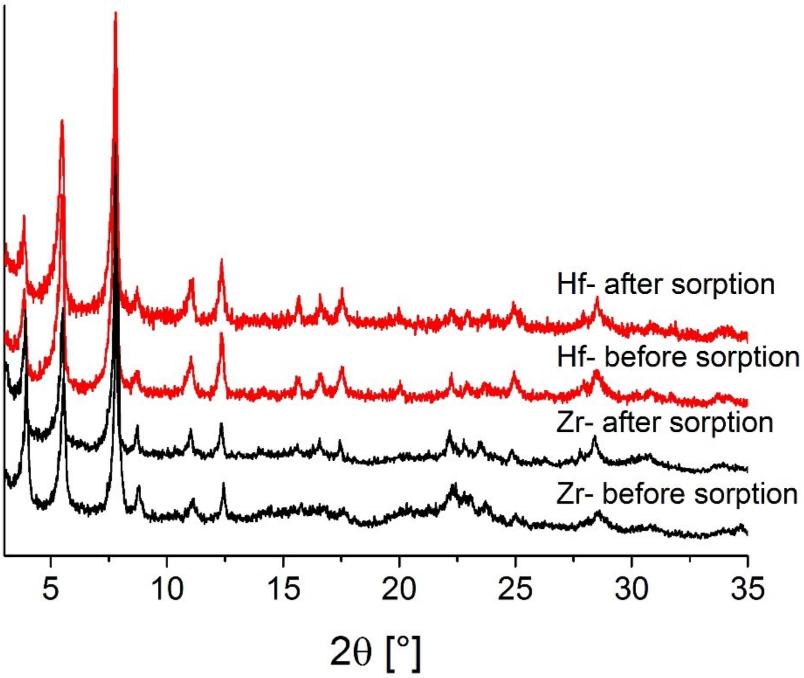

Figure S9. PXRD patterns of M-CAU-30 (M= Zr, Hf) after the sorption experiments (activation 120 °C, 10^{-2} kPa). For comparison the patterns of M-CAU-30 before the sorption experiment are also presented.

4. TG investigations, PXRD patterns of the decomposition products, VT-PXRD studies and chemical stability.

The results of the TG measurement of Hf-CAU-30 are shown in Fig. S10 and Tab. S3. The decomposition products of M-CAU-30 (M= Zr, Hf) mostly consists of Ni-M-phosphates and MO_2 which was shown by PXRD measurements (Fig. S11). The results of VT-PXRD studies (Fig. S12) confirm the results obtained from the TG measurements. Complete collapse of the frameworks takes place above ca. 400 °C.

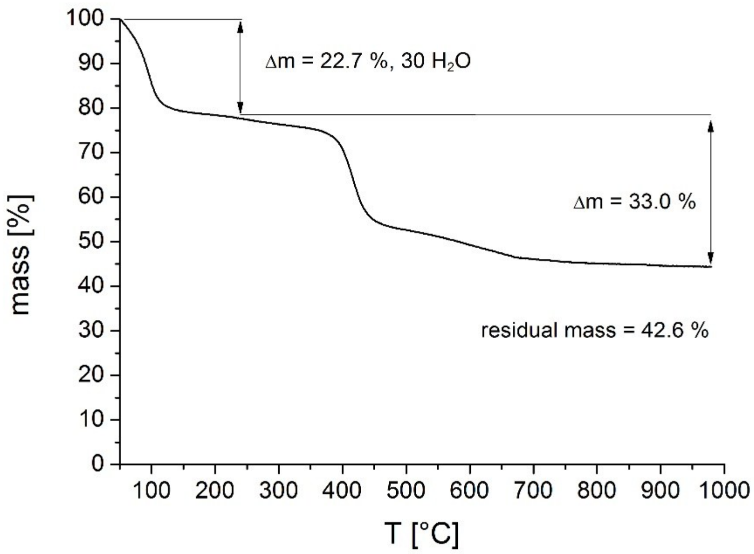

Figure S10. Thermogravimetric curve of Hf-CAU-30 $[Hf_2(Ni\text{-}H_2TPPP)(OH/F)_2] \cdot 30\ H_2O$ (Table S4).

Table S3. Results of the thermogravimetric measurements of M-CAU-30 (Zr, Hf) calculated for 0 and 1 MO_2 per formula unit.

sum formula	residual mass<br>meas. [%]<br>calc. [%]	mass loss 200-1000 °C<br>linker decomposition<br>meas. [%]<br>calc. [%]	mass loss<br>25-200 °C H_2O<br>meas. [%]<br>calc. [%]
$[Zr_2(Ni\text{-}H_2TPPP)(OH/F)_2]$<br>$\cdot 23\ H_2O$	38.2<br>33.2	39.6<br>44.0	20.5<br>20.5
$[Zr_2(Ni\text{-}H_2TPPP)(OH/F)_2]$<br>$\cdot 23\ H_2O \cdot ZrO_2$	38.2<br>37.4	39.6<br>41.2	20.5<br>20.5
$[Hf_2(Ni\text{-}H_2TPPP)(OH/F)_2]$<br>$\cdot 30\ H_2O$	42.6<br>36.5	33.0<br>37.6	22.7<br>22.7
$[Hf_2(Ni\text{-}H_2TPPP)(OH/F)_2]$<br>$\cdot 30\ H_2O \cdot HfO_2$	42.6<br>42.2	33.0<br>34.2	22.7<br>22.7

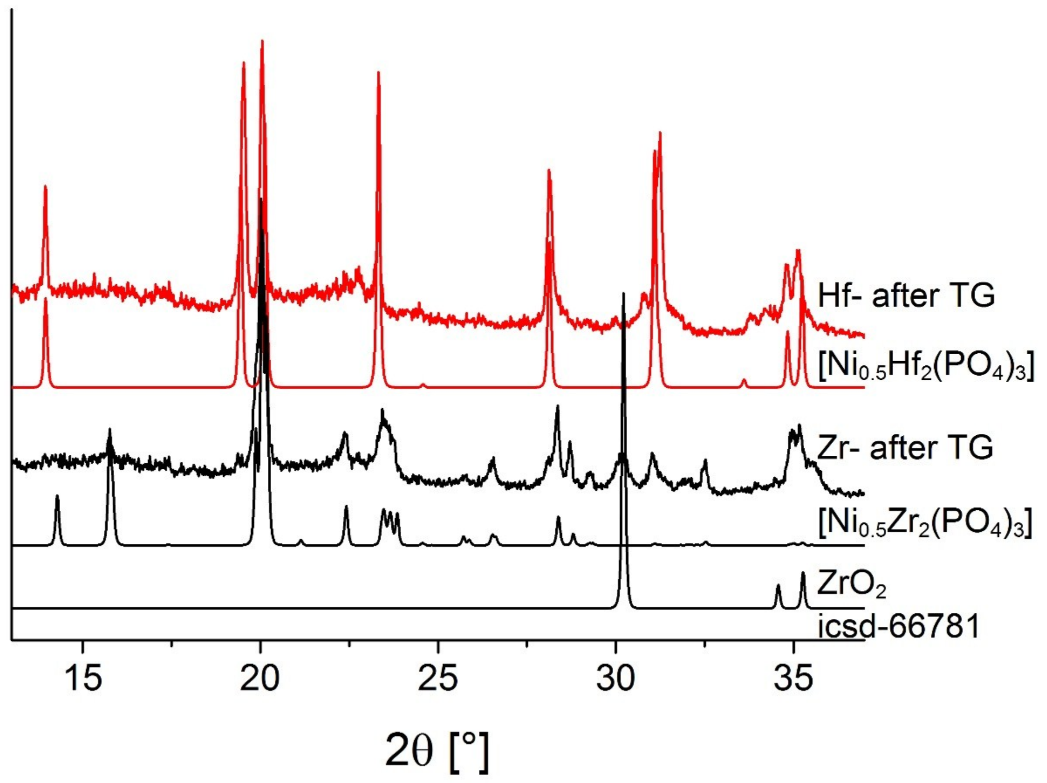

Figure S11. PXRD patterns of M-Ni-phosphates and oxides (M=Zr, Hf) compared with the residual products of the thermogravimetric experiments of M-CAU-30.

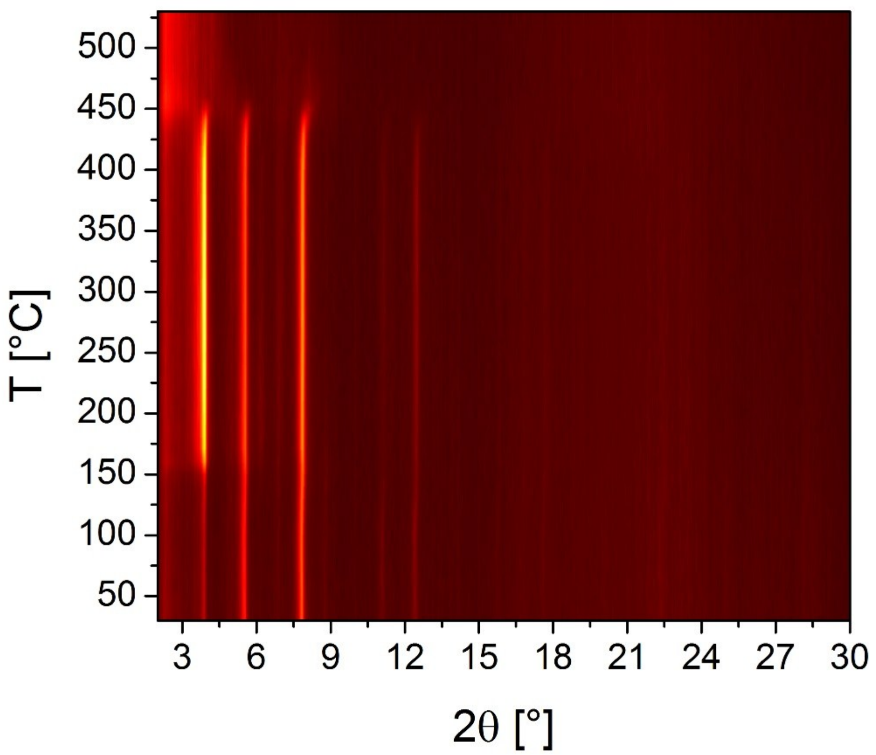

Figure S12. Results of the VT-PXRD studies of Hf-CAU-30 (Cu-Kα_1 radiation) measured in open quartz capillaries (0.5 mm).

To study the chemical stability M-CAU-30 were stirred in different solvents for 24 h and subsequently characterized by PXRD measurements (Fig. S13). Both MOFs are stable in all tested organic solvents as well as in a pH range between 0 and 12 (aqueous HCl/ NaOH solutions). Furthermore both MOFs are stable in 0.1 M phosphate buffer. Hf-CAU-30 shows a loss of the long range order by stirring in 100 % acetic acid, while Zr-CAU-30 is stable.

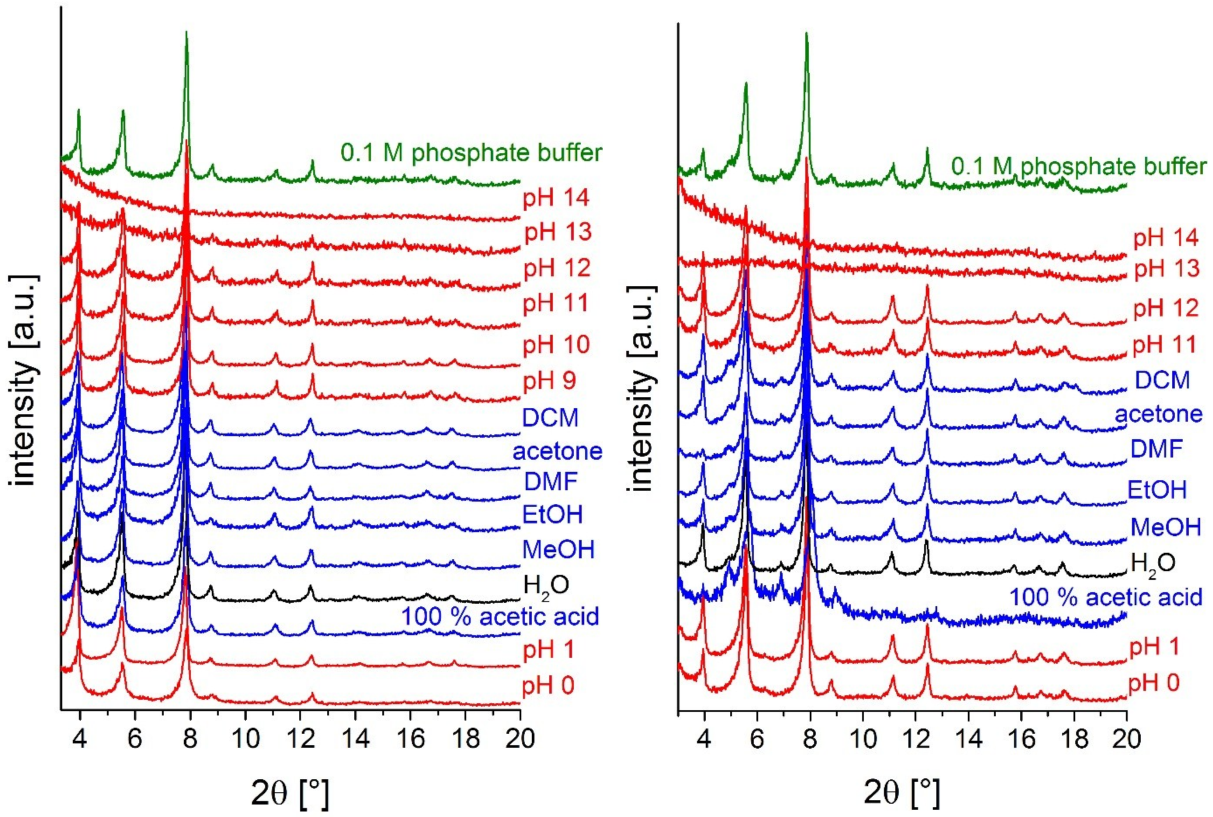

Figure S13. Chemical stability of Zr-CAU-30 (left) and Hf-CAU-30 (right) in different solvents (24 h, stirring at room temperature).

5. Spectroscopy

IR spectra of M-CAU-30 (M= Zr, Hf) and the Ni-H_8TPPP linker are shown in Figure S14. The assignment of the bands are given in Table S4.

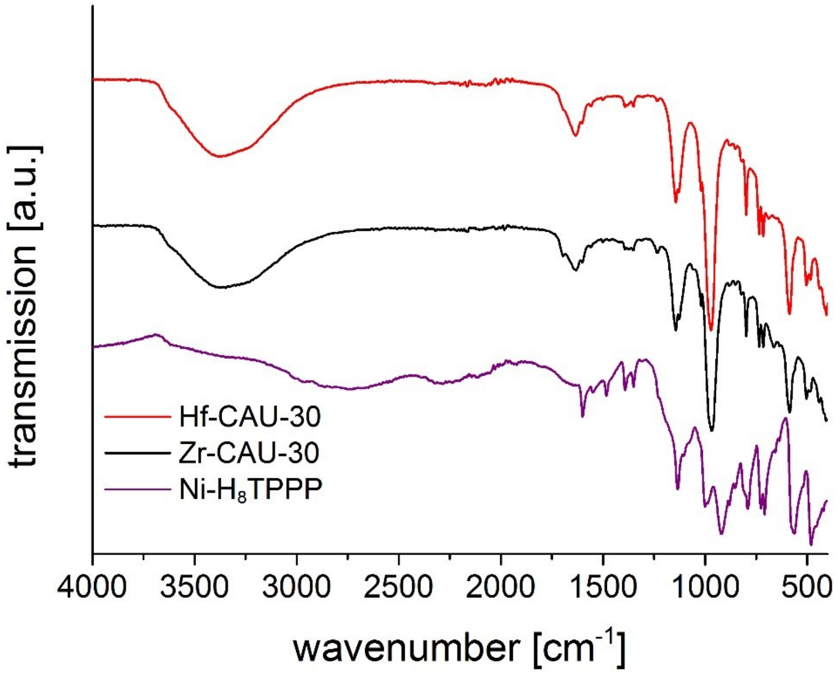

Figure S14a. IR spectra of M-CAU-30 (M=Zr, Hf) compared with the IR spectrum of the free linker Ni-H_8TPPP.

Table S4. Assignment of the vibrations in the IR-spectra of M-CAU-30 (M= Zr, Hf) and the free linker Ni-H_8TPPP.[11, 12]

Vibration $\tilde{\nu}$IR [cm^{-1}]	Zr	Hf	Ni-H_8TPPP
ν OH (H_2O)	3389	3389	
ν P-OH	1634	1634	1606
ν C=C (arom.)	1562	1562	1519
ν P-C (phenyl)	1501	1501	1480
δ C=C, N=C (in plane)	1392	1392	1389
ν C-N (pyrrole)	1351	1351	1352
ν P=O (stretching)	1234	1234	1223
ν P-O (stretching)	1141	1141	1136
δ C-H, N-H (pyrrole)	over	layed	1100
δ C-H (in plane)	over	layed	1005
ν P=O (stretching)	966	966	921
γ C-H (1,4-subst.)	800	800	793
δ C-H, N-H (pyrrole)	737	737	730
γ P-C	717	717	704
δ $P(OR)_3$	588	588	567
δ C=C (skeleton)	506	506	484

6. TEM and SEM measurements

Scanning Electron Microscopy (SEM)

All SEM micrographs shown were acquired using a *Helios NanoLab G3UC* (*FEI*) electron microscope operating at 3 keV. Sample preparation was performed by dispersing both Zr-CAU-30 and Hf-CAU-30 in ethanol via sonication and subsequently drying respective aliquots on a carbon film placed on an aluminum sample holder. Before performing the actual measurements the samples were sputtered with carbon. For data evaluation the software *ImageJ v1.49.* was used.

Transmission Electron Microscopy

TEM micrographs and EDX investigations were recorded either on a *Tecnai G2 20 S-TWIN (FEI)* or a *Titan Themis 60-300* (*FEI*) electron microscope that were operated at an acceleration voltage of 200 kV and 300 kV, respectively. For sample preparation, ethanolic dispersions of Zr-CAU-30 and Hf-CAU-30 were prepared and then dried on a carbon-coated copper grid. For data evaluation the software *ImageJ v1.49.* was used.

Zr-CAU-30

To further analyze the spherical secondary phase visible in the STEM images in Figure 5, electron diffraction was performed on the particles: Figure S15 (left) shows a typical area of the aggregated and intergrown nanoparticles along with a diffraction pattern in Figure S15 (right). Due the small domain sizes the reflections of the nanocrystals are broadened.

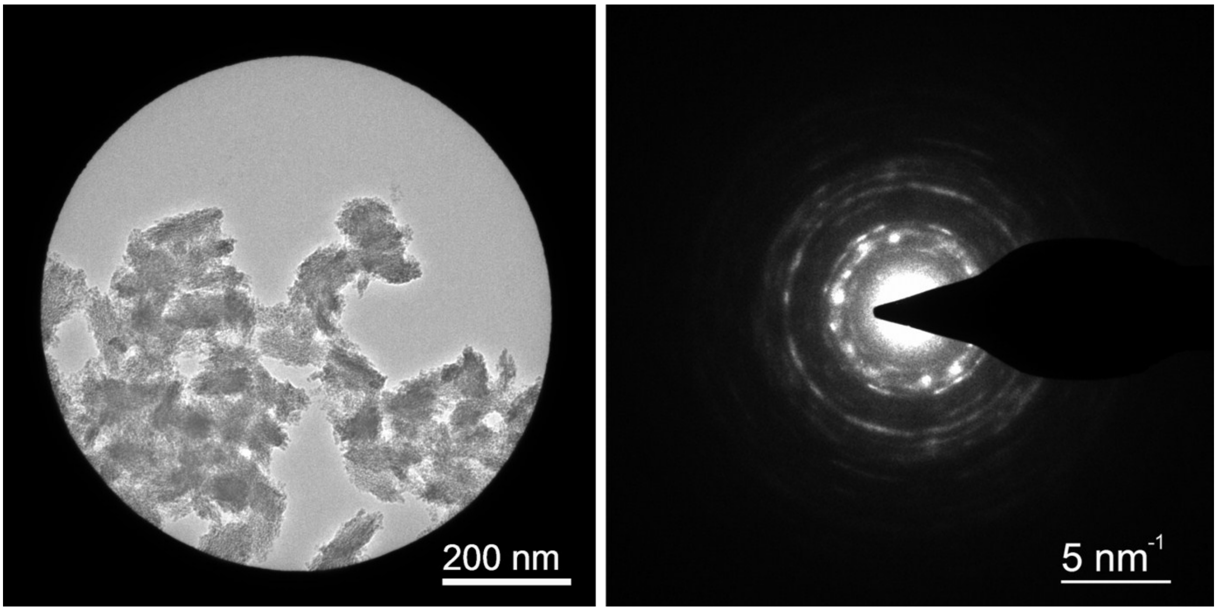

Figure S15: Secondary phase observed in the reaction produce that led to the formation of Zr-CAU-30 along with the respective electron diffraction pattern.

The most prominent lattice distances are listed in

Bragg Ring (Inside to Outside)	Lattice distance
1	0.36 nm
2	0.30 nm
3	0.26 nm
4	0.20 nm
5	0.18 nm
6	0.17 nm

5.They are in good agreement with the monoclinic phase of ZrO_2.[13]

Table S5: Lattice distances of secondary phase in Zr-CAU-30.[13]

Bragg Ring (Inside to Outside)	Lattice distance
1	0.36 nm
2	0.30 nm
3	0.26 nm
4	0.20 nm
5	0.18 nm
6	0.17 nm

To further identify the constituents of this phase, EDX measurements were performed on the nanoparticles. The area chosen for the measurements is shown in **Figure S1S16**. The results of these measurements are summarized in **Table S6**, excluding the support film material carbon. The two largest fractions are Zirconium and Oxygen while both nickel and phosphor are absent. This shows that this secondary phase does not contain any linker molecules and is in agreement with the presence of zirconium oxide as determined by electron diffraction.

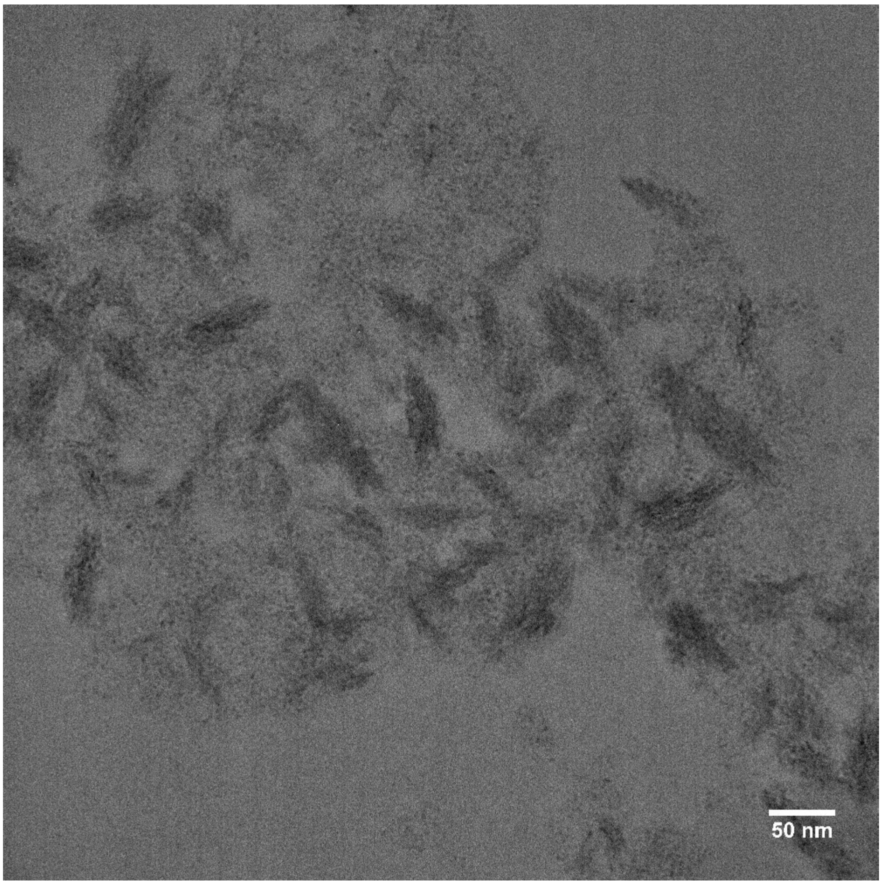

Figure S16: Secondary phase observed in the reaction produce that led to the formation of Zr-CAU-30.

Table S6: Composition of secondary phase observed in the reaction produce that led to the formation of Zr-CAU-30 derived from EDX measurement, excluding carbon. The first column refers to the element and the x-ray line used for quantification.

Element	Atomic %
Zr(L)	24.2
O(K)	69.2
Na(K)	3.0
F(K)	3.5

Hf-CAU-30

In a similar manner to Zr-CAU-30, Hf-CAU-30 was also examined by TEM. An overview micrograph, depicting the morphology of the species is shown in Figure S17. Similar to Zr-CAU-30, the majority of Hf-CAU-30 consists of needles that are up to 700 nm long with a width of 30 - 60 nm. In addition to that two other morphologies are present in the sample as shown in **Fehler! Verweisquelle konnte nicht gefunden werden.** Next to the needles there are spherical aggregations of particles with a diameter between 2 – 5 nm and a cube-shaped crystalline species featuring particle diameter between 40 – 60 nm. Both needles and cube-shaped species are crystalline as can be seen on the periodic lattice planes visible in Figure S19.

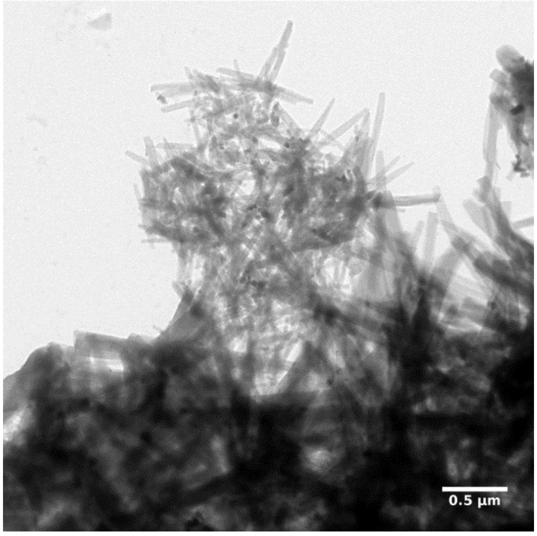

Figure S17: TEM micrograph of Hf-CAU-30.

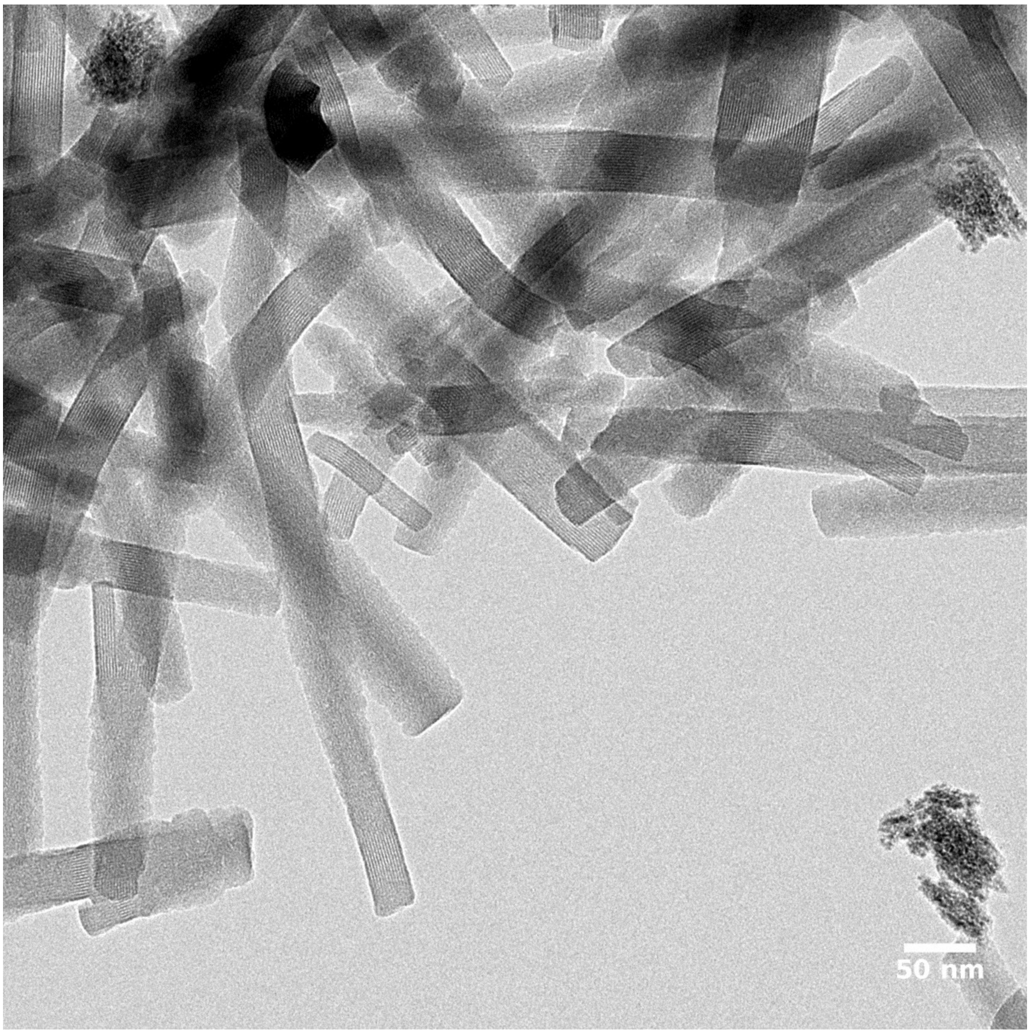

Figure S19: Crystalline Needles of Hf-CAU-30

Nanocrystalline aggregates in the Hf-CAU-30 sample

In addition to the needle-like and the cubic morphologies observed in this sample, nanocrystalline aggregates were observed as well, similar to the secondary phase in the Zr-CAU-30 sample. The particles with their corresponding diffraction pattern are shown in Figure S20. The observed lattice plane distances (Table S7) are very similar to those observed for the secondary phase in the Zr-CAU-30 sample, albeit slightly smaller. This is consistent with a HfO_2 phase isostructural to monoclinic ZrO_2 but having slightly smaller lattice parameters, as known from the literature.[13]

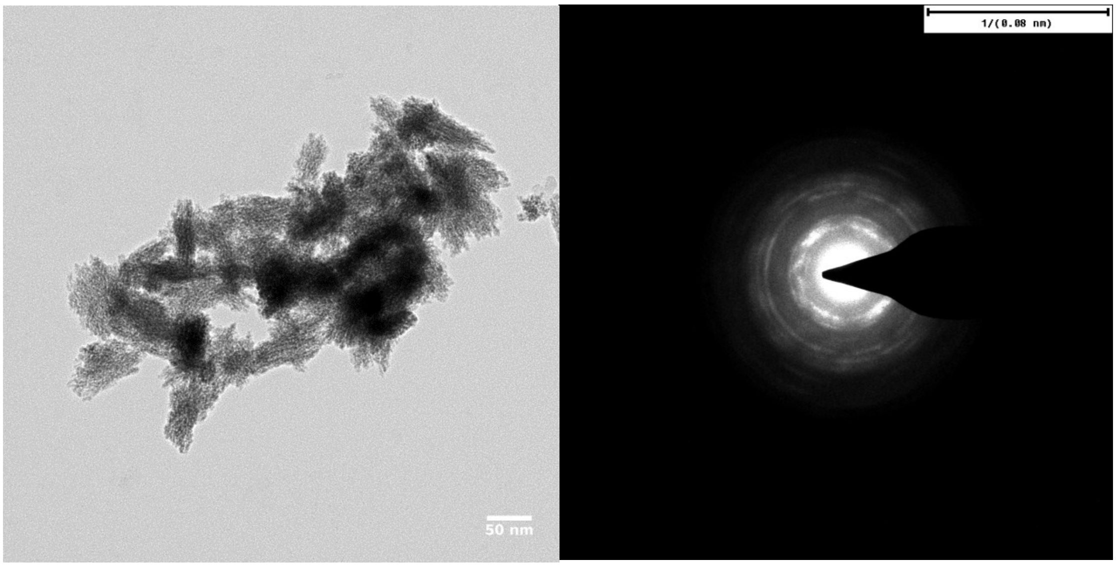

Figure S20: Secondary phase of sample Hf-CAU-30 along with electron diffraction pattern. (Bragg rings are marked with yellow lines)

Table S7: Lattice plane distances of secondary phase in Hf-CAU-30.[13]

Reflection	Lattice plane distance
1	0.35 nm
2	0.25 nm
3	0.18 nm
4	0.17 nm
5	0.13 nm

Evaluations of EDX spectra of Hf-CAU-30 and the secondary phase are shown in Table S8. The elemental quantifications confirm the expected respective compositions of Hf-CAU-30 and HfO_2. The presence of sodium and fluorine is a result of the synthesis route employing NaOH and NaF.

Table S8: Results of EDX measurements of Hf-CAU-30. Both quantifications exclude carbon, the substrate film material. The first column refers to the element and the X-ray line used for quantification. The second and the third column respectively refer to the two phases observed, easily to distinguish by their morphologies.

	Hf-CAU-30 [at %]	Secondary phase [at %]
Hf (L)	7.0	28.0
O (K)	58.2	63.2
Ni (K)	2.7	1.0
N (K)	14.3	0.0
P (K)	11.3	4.3
Na (K)	5.5	2.6
F (K)	1.0	0.9

7. Cyclic voltammetry

Cyclic voltammograms (CVs) of Zr-CAU-30 were measured on a flour doped tin oxide (FTO) electrode in 0.1 M phosphate buffer (pH 7) with an Ag/AgCl type reference electrode and Pt as counter electrode. The cyclic stability was demonstrated by measuring 50 cycles; the first and the 50th CV is shown in Figure S22. Applying different scan rates between 50 and 1000 mV s-1 reveals the expected behavior of increasing currents with the scan rate (Fig. S23).

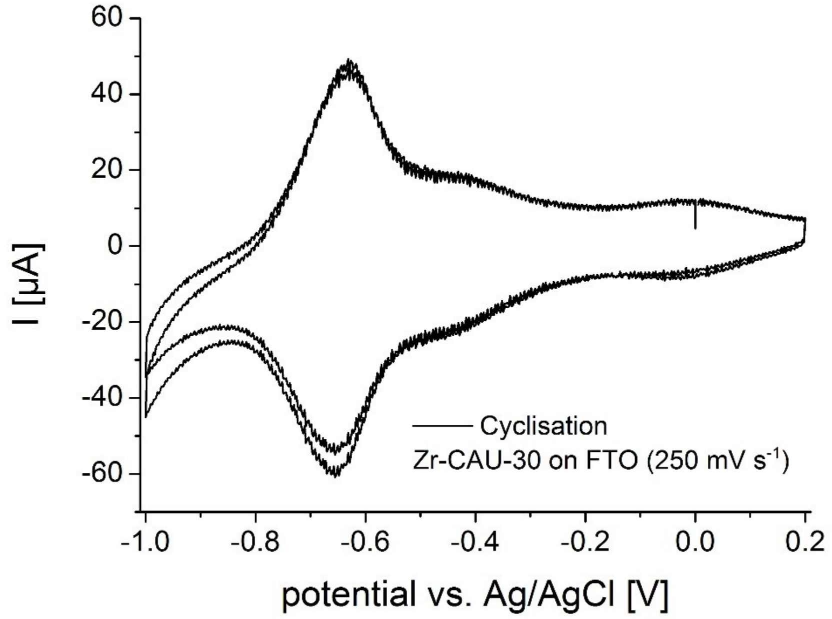

Figure S22: Results of the cyclic stability of Zr-CAU-30 on FTO. The CVs of the first and 50th cycle indicate no significant decrease in the measured current.

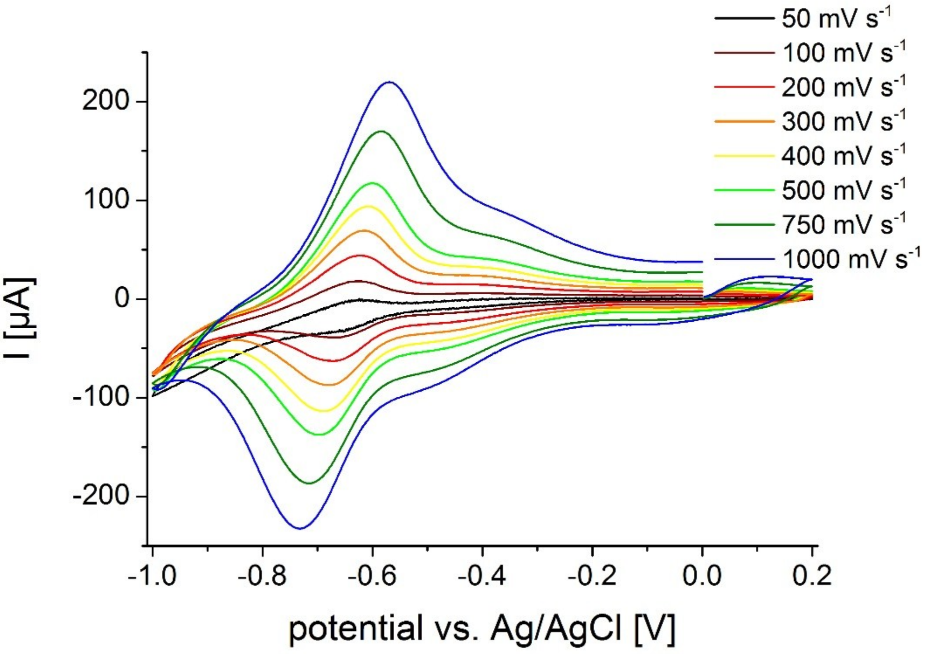

Figure S23. CVs of Zr-CAU-30 on FTO at different scan rates between 50 and 1000 mV s^{-1}.

8. References

1. M. Taddei, F. Costantino and R. Vivani, *Inorg. Chem.*, 2010, **49**, 9664-9670.
2. M. Taddei, F. Costantino, F. Marmottini, A. Comotti, P. Sozzani and R. Vivani, *Chem. Commun.*, 2014, **50**, 14831-14834.
3. M. Taddei, F. Costantino, R. Vivani, S. Sabatini, S.-H. Lim and S. M. Cohen, *Chem. Commun.*, 2014, **50**, 5737-5740.
4. R. K. Mah, B. S. Gelfand, J. M. Taylor and G. K. H. Shimizu, *Inorganic Chemistry Frontiers*, 2015, **2**, 273-277.
5. C.-Y. Gao, J. Ai, H.-R. Tian, D. Wu and Z.-M. Sun, *Chem. Commun.*, 2017, **53**, 1293-1296.
6. T. Zheng, Z. Yang, D. Gui, Z. Liu, X. Wang, X. Dai, S. Liu, L. Zhang, Y. Gao, L. Chen, D. Sheng, Y. Wang, J. Diwu, J. Wang, R. Zhou, Z. Chai, T. E. Albrecht-Schmitt and S. Wang, *Nat Commun.*, 2017, **8**, 15369.
7. A. D. Adler, F. R. Longo, F. Kampas and J. Kim, *J. Inorg. Nucl. Chem.*, 1970, **32**, 2443-2445.
8. A. D. Adler, F. R. Longo, J. D. Finarelli, J. Goldmacher, J. Assour and L. Korsakoff, *The Journal of Organic Chemistry*, 1967, **32**, 476-476.
9. G. G. Rajeshwaran, M. Nandakumar, R. Sureshbabu and A. K. Mohanakrishnan, *Org. Lett.*, 2011, **13**, 1270-1273.
10. P. Tavs, *Chem. Ber.*, 1970, **103**, 2428-2436.
11. G. Socrates, *Infrared and Raman Characteristic Group Frequencies: Tables and Charts*, Wiley, 2004.
12. M. Hesse, H. Meier and B. Zeeh, *Spektroskopische Methoden in der organischen Chemie*, Thieme, 2005.
13. R. E. Hann, P. R. Suitch and J. L. Pentecost, *J. Am. Ceram. Soc.*, 1985, **68**, C-285-C-286.
